# Initial validity and reliability testing of the SGBA-5

**DOI:** 10.1371/journal.pone.0323834

**Published:** 2025-05-16

**Authors:** Andrew Putman, Adam Cole, Shilpa Dogra

**Affiliations:** Faculty of Health Sciences, Ontario Tech University, Oshawa, Ontario, Canada; Ministry of Health, Sri Lanka, SRI LANKA

## Abstract

**Background:**

A growing body of research indicates that sex (biological) and gender (sociocultural) influence health through a variety of distinct mechanisms. Sex- and Gender-Based Analysis (SGBA) techniques could examine these influences, however, there is a lack of nuanced and easily implementable measurement tools for health research. To address this gap, we created the Sex- and Gender-Based Analysis Tool – 5 item (SGBA-5).

**Objectives:**

This research aims to assess the validity and reliability of the SGBA-5 for use in health sciences research where sex or gender are not primary variables of interest.

**Methods:**

A Delphi consensus study was conducted with Canadian researchers (n = 14). The Delphi experts rated the validity of each SGBA-5 item on a 5-point Likert scale each round, receiving summary statistics of other experts’ responses after the first round. A conservative threshold for consensus agreement (75% rating an item 4+ of 5) was used given the novelty of this scale’s items. Reliability was assessed through a two-armed test-retest study. The university student arm (n = 89) was conducted in-person (on paper), and the older adult arm (n = 71) was conducted online (digitally).

**Results:**

The Delphi study ended after three rounds; experts reached consensus agreement on the validity of the biological sex item of the SGBA-5 (93%) and consensus non-agreement on each of the gendered aspect of health items (identity: 64%, expression: 64%, roles: 50%, relations: 57%). Both the student arm (sex item: $\begin{array}{c} \kappa =1.00,95\\ \%CI(1.00,1.00) \end{array}$, gendered items: $\begin{array}{c} {ICC}_{\left(A,1\right)}\geq \mathrm{.899},95\\ \%CI(\mathrm{.851},\mathrm{.933}) \end{array}$) and the older adult arm (sex item: $\begin{array}{c} \kappa =1.00,95\\ \%CI(1.00,1.00) \end{array}$, gendered items: $\begin{array}{c} {ICC}_{\left(A,1\right)}\geq \mathrm{.865},95\%CI(\mathrm{.772},\mathrm{.920}) \end{array}$) of the test-retest study indicated that all items were reliable.

**Conclusions:**

The novel SGBA-5 tool demonstrated reliability across all scale items and validity of the biological sex item. The gendered aspects of health items may be valid. Future research can further develop the SGBA-5 as a tool for use in health research.

## Introduction

In health sciences research, Sex- and Gender-Based Analysis (SGBA) is an umbrella term for the collection of research methods and analytic techniques that can provide insight into how sex and gender interact with health. In this context, sex is defined as biological characteristics in living beings that relate to sexual reproduction, and gender is defined as the socio-cultural expectations, roles, expressions, and identities that are associated with women, men, and gender diverse people [1]. Many leading organizations - including the Canadian tri-council funding agencies, the National Institutes of Health (US), the European Association of Science Editors, and the World Health Organization - have encouraged and prioritized systematic inclusion of SGBA. In fact, all of them have created specific policies promoting the inclusion of SGBA in health research [2–9].

Despite these collective efforts, the integration of SGBA into health research has not been universal. For example, in 2009 the Canadian Institutes of Health Research (CIHR) began implementation of their 10-year SGBA action plan; this plan required that all grant applicants had to complete SGBA training modules prior to submitting their applications [2,10,11]. Haverfield and Tannenbaum (2021) analyzed more than 39,000 grant applications to the CIHR from 2011 to 2019 and found that while the inclusion of sex-based analysis increased from 22% of grant applications in 2011 to 83% in 2019, the inclusion of gender-based analysis went from 12% in 2011 to 33% in 2019 [10]. The relatively small increase in the inclusion of gender-based analysis into grant applications suggests that researchers are finding it harder to integrate gender-based analysis into their study designs than sex-based analyses. It was further noted that despite completing the required SGBA training modules, a portion of both the grant applicants and the grant evaluators demonstrated a lack of comprehension of core SGBA principles, such as conflating sex and gender [10]. This gap between the goal of SGBA implementation by funding agencies and research entities, and the integration by researchers in individual studies and grant applications is influenced by many factors, including the limited number of valid and reliable tools that can be used for SGBA.

Several measurement tools have been created over the past 50 years that aim to assess the effects of sex and/or gender in a health context. However, there are several limitations to these measurement tools. Common limitations are that these tools can be lengthy, invasive, overly reliant on stereotypes, offensive, demeaning, or based on outdated conceptions of sex and gender [6,12,13]. A more detailed analysis of strengths and limitations of existing tools is available elsewhere [14]. More specifically, there is currently a lack of valid and reliable tools that are concise while allowing for differentiation between biological sex and gender as a social determinant of health in research where sex or gender are not the primary focus. For example, in 2022 the National Academy of Sciences (US) recommended that researchers use a two-step method of nominal categorical responses where participants report their sex-assigned at birth and gender identity separately [15]. The recommendation of the two-step method is supported by evidence of its validity and reliability for use in population-level and census questionnaires [15–17]. Unfortunately, these kind of categorical indicator variables can only provide meaningful differentiation between sex-based and gender-based effects in studies that have very large sample sizes, an inherent limitation of using disaggregation analyses. Disaggregation-based analyses do not allow for the level of detailed insight that is possible from more complex conceptualizations of sex and gender – which require more intricate scale measures or qualitative work [18]. Thus, the use of nominal categories and disaggregated analysis in SGBA can provide some insight into differences between sex and/or gender categorizations in large, population-scale surveys, they cannot provide the level of granular detail needed for investigation of the multidimensional aspects and within-category variation that is inherent in social constructs like gender [6].

To address this gap, we created the *Sex- and Gender-Based Analysis Tool – 5 Item* (SGBA-5). This tool is proposed as *one way* to conduct SGBA in health research studies based on current evidence of how sex and gender influence health but is certainly not *the* *only way* to do so. There are a multitude of ways to model and conceptualize sex and gender, and how they impact health; the SGBA-5 is one of many such tools that are needed to address the breadth of potential SGBA implementations in the health sciences [2,6,12]. Thus, the purpose of this study was to assess the validity and reliability of the SGBA-5 for use in health sciences research where sex or gender are not primary variables of interest.

## Methods

**Fig 1:**
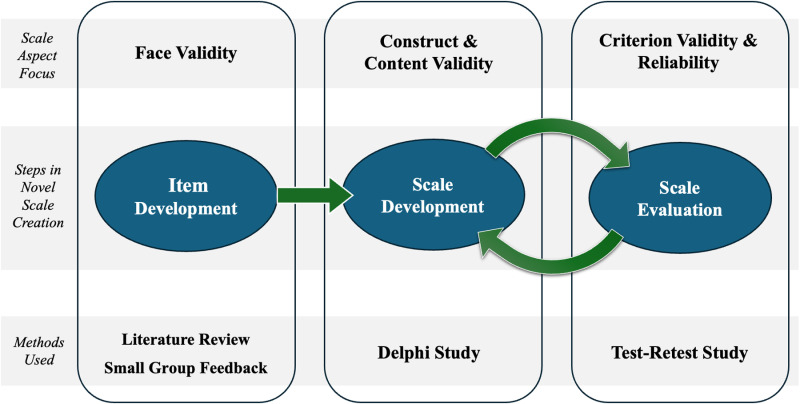
Visual representation of the cyclical process of novel scale development. This diagram shows the steps involved in novel scale creation, beginning with Item Development and then progressing to an ongoing cycle of Scale Development and Scale Assessment. This cycle of Scale Development and Scale Assessment continues alongside use of the scale to ensure the scale’s validity and reliability as well as to assess the suitability of the scale’s use in different contexts. The diagram also indicates the steps of novel scale development associated with the initial design and testing of the SGBA-5 described in this paper.

The first iterations of the SGBA-5 were developed alongside a thorough literature review and small-group feedback. The more formal assessment of the SGBA-5 began with a Delphi expert consensus study on the SGBA-5’s validity, and then continued with a test-retest study to assess its reliability. These steps and their relation to the steps in creation of a novel measurement tool (scale) are visualized in Fig 1 [18–20].

### Initial design and development

As is typical with the development of a scale that does not have direct comparator scales to draw upon, potential items for inclusion in the SGBA-5 were drawn from extensive reviews of the peer-reviewed and grey literature relating to biological sex, gendered aspects of health, measurement of multidimensional health impacts, continuous scale creation, and scale validation methodologies [8,12,15,18,19,21–29]. One of the key takeaways from this earlier investigation was that at present, while there is theoretical backing for a variety of ways in which to operationalize measurement of aspects of gender that may influence health [2,6,12], there is not a similar theoretical background for the measurement of different aspects of biological sex in general health research.

The SGBA-5 consists of a categorical option for biological sex (male/female/intersex) and four gendered aspect of health constructs measured on a visual analogue scale that depicts a feminine-masculine continuum. The wording and type of measurement for each item that is included in SGBA-5 is presented in Table 1 and is included in S1 File. The categorical option of biological sex was selected as the authors could not find a theoretical foundation on which to base a more complex understanding of biological sex that is both appropriate for use in a general population or that could be answered on a questionnaire without *a priori* knowledge of specific biological test results. A short list of potential gendered aspects of health to include in the SGBA-5 was derived from a selection of the existing academic literature, white papers, and SGBA policies that was identified in the initial background literature review [2,6,8,12,13,26]. The four gender constructs included in the SGBA-5 were chosen because evidence was found in the literature to support their proposed pathway of health impact (gender identity [30,31], gender expression [32,33], gender roles [34,35], and gendered relations[36,37]). Institutionalized gender as a gendered aspect of health was considered but not included in the SGBA-5 as institutional-level impacts are best assessed at a legislative or community level rather than on an individual level [6,12].

**Table 1:** Description of the items included in the SGBA-5.

Item	Item Wording	Type of Measure	Possible Responses
Biological Sex:			
Sex	*“Please indicate your sex assigned at birth:”*	Nominal Categories	Male, Female, Intersex
Gendered Aspects of Health:			
Identity	*“How would you generally describe yourself on a feminine to masculine continuum?”*	Continuous (Analogue Scale)	0 – 100^a^^,^^b^
Expression	*“How would you describe your typical behaviour and mannerisms on a feminine to masculine continuum?”*	Continuous (Analogue Scale)	0 – 100^a^^,^^b^
Roles	*“How would you describe your day-to-day responsibilities on a feminine to masculine continuum?”*	Continuous (Analogue Scale)	0 – 100^a^^,^^b^
Relations	*“How would you generally describe how your friends, colleagues, and authority figures in your life typically treat you on a feminine to masculine continuum?”*	Continuous (Analogue Scale)	0 – 100^a^^,^^b^

^a^Item is presented using a feminine – masculine continuum with the only markings being the feminine and masculine anchors at either end of the line. *On paper:* recorded with a 100mm line. *Digitally:* recorded with 101-point slider.

^b^See example analysis at https://github.com/putman-a/SGBA-5_example_analysis [38] for more detailed explanation of interpretation.

As the CIHR Institute of Gender and Health notes in its definitions of sex and gender in the context of health research, “[gender] is not confined to a binary (girl/woman, boy/man) nor is it static; it exists along a continuum and can change over time” [1]. To attempt to capture more of this variation than nominal items alone could provide, feminine – masculine analogue (continuous) scales were used to represent the four gendered aspects of health constructs that were included in the SGBA-5; they are not assumed to have true zero [0] values. We implemented this interpretation constraint onto the SGBA-5’s analogue measures with the aim to mitigate potential over-interpretation of differences between groups or individuals who complete the SGBA-5 as a part of a health study where sex or gender are not primary variables of interest. Despite the limitations of assuming that the analogue measures do not have a true zero value, these measures can still provide more information on group and individual-level variation than would be possible from a nominal categorization item alone [18].

We aimed to design a tool for within study analysis of the impacts that sex or gender have on the study’s primary outcomes. **The SGBA-5 is not designed to ‘determine’ any individual participant’s sex or gender, nor is designed to be sensitive enough for research where sex or gender are the primary variable[s] in the study.** The SGBA-5 is not a replacement for dedicated work with marginalized sex and gender communities (i.e., trans & nonbinary individuals, intersex persons, etc.) however, the SGBA-5 is designed so that it can be completed by persons of sex and gender minorities as part of studies where sex or gender are not primary variables. The SGBA-5 is designed with the intention to be integrated into clinical trials and research studies, enabling researchers to facilitate the inclusion of multiple dimensions of SGBA into their studies. Completing the SGBA-5 requires one to two minutes, and thus should not be onerous for the participant or researcher to use. Additional information on scale methodology rationale, appropriate application, and interpretation of the SGBA-5 are also included in S1 File.

After developing the first full version of the SGBA-5, we informally presented it to health sciences researchers at a Faculty seminar (n ≈ 30), and at a multi-university journal club meeting (n ≈ 25) to obtain feedback on the face validity of the SGBA-5. The five selected item topics and their respective methods of measurement remained consistent throughout the small-group feedback stage of development, but the presentation and phrasing of those items were updated and iterated upon throughout.

### Delphi expert consensus

More rigorous initial evaluation of the SGBA-5’s suitability for use in health research involved a Delphi expert consensus of Canadian health researchers to assess the content validity of the SGBA-5 for within-sample SGBA. Generally, a Delphi study of content validity consists of a minimum of three rounds in which experts independently evaluate the proposed scale item and score it using a Likert scale or pass/fail rating [19,39]. These evaluations are communicated to the Delphi researchers who pool and assess the expert feedback. From the second round onward, the researchers typically provide the Delphi experts with descriptive statistics and/or qualitative summaries from the previous round’s anonymized expert feedback [40,41]. This anonymized feedback provides experts an overview of the other Delphi experts’ opinions which they can use to inform their evaluation of that round. A Delphi study is halted once the experts reach a stable consensus, or if a pre-determined maximum number of rounds has been reached (not used in this study), or if the between-round differences in the Delphi expert’s rating drops below a predetermined threshold (used in this study) [39–41].

The purpose of this Delphi Expert Consensus study was to receive feedback on the construct validity of the SGBA-5’s scale items from a sample of Canadian health sciences researchers (this study’s Delphi experts and the most likely initial users of the SGBA-5). This process provided evidence that each item measures what it is proposed to measure (an item’s content validity) [18,19]. In accordance with the threshold of evidence used to initially select the items for inclusion in the SGBA-5, it was decided *a priori* that any major changes (i.e., adding a new item, switching from continuous to ordinal measures, etc.) resulting from the Delphi expert’s feedback must reflect evidence in the current health sciences literature.

### Participants

Beginning January 10^th^, 2023, the authors (AP and SD) contacted health science Deans at institutions across Canada and requested that they recommend 1–2 potential participants. Specifically, we asked for them to identify researchers who had expertise in conducting health research studies with human participants, and who had been involved in CIHR-funded research in the past (to ensure familiarity with the CIHR sex and gender definitions in health research). By the end of participant recruitment on Feb 14^th^, 2023, 32 researchers had been recommended to the authors as potential experts for the Delphi, 17 of whom consented to participate in the study. Fourteen experts (82%) participated in all three rounds. The Delphi experts represented institutions spanning seven provinces and one territory. All participants provided written consent prior to participation in the study. The Delphi study was reviewed by and conducted in compliance with the regulations of the Ontario Tech University Research Ethics Board (REB # 17153).

### Procedure

In the first round, participants were emailed a link to a survey in which they were presented each of the items from the SGBA-5 as well as the instruction page that would be provided to researchers administering the SGBA-5. Participants were asked to score each scale item from the SGBA-5 on a 1–5 Likert scale, with a score of 1 being “This question is not a valid measure of [scale item] for SGBA in health research”, and a score of 5 being “This question is a valid measure of [scale item] for SGBA in health research”. The participants were also able to provide optional written feedback on the scale items’ construct validity individually, or the SGBA-5 as a whole.

In the second and third rounds, participants were asked to conduct the same rating exercise and were also provided with optional supplementary documentation that further explained the rationale for the SGBA-5, as well as a short Question & Answer-style summary of the SGBA-5’s creation, and appropriate use cases. In these rounds, participants were shown the median and interquartile ranges of the Likert scores from the previous round when rating each scale item. Furthermore, minor adjustments were made to the SGBA-5’s formatting or phrasing between rounds based on comments from the previous round.

### Statistical analysis

The threshold for consensus agreement was set *a priori* to 75%. That is, the expert rating of each item from the SGBA-5 had to be: 1) rated at least 4 out of 5 on the Likert scale, and 2) have a $X^{2}$ test demonstrating inter-round answer stability at α=.05 after a minimum of three rounds. Alternatively, the Delphi study would also be halted if the between-round difference in the coefficient of variance (ΔCV) was <.15, which would be an indication that the researchers have reached a stable non-agreement consensus. These thresholds are in line with Delphi Expert Consensus best practices and intentionally use more conservative target measures to avoid overestimation of expert consensus [18,19,39–41].

The statistical tests for consensus ($\backslash \%\geq 4of5,X^{2},\Delta CV$) and overall summary statistics (median, IQR) from and between each round were calculated after the completion of each round in the Delphi study.

### Test-retest study

A test-retest component aimed to assess the reliability of each scale item in the SGBA-5 in two populations.

### Participants

The test-retest reliability of the SGBA-5 was assessed in two separate populations, university students and older adults. These two populations were recruited and assessed separately in an in-person student arm, and a virtually administered older adult arm.

Participants in the student arm were recruited from the student participant pools of the Kinesiology and Psychology programs at Ontario Tech University between September 4^th^, 2023, and November 13^th^, 2023. Inclusion was limited to those who were a current student at Ontario Tech University, those able to come to two in-person sessions, and those able to communicate in English. The older adult arm was recruited between September 11^th^, 2023, and February 2^nd^, 2024 through Ontario Tech University’s Age-Friendly Campus email newsletter and through Facebook advertisements targeting older adults in the Durham Region. Participants were eligible for the older adult arm if they were 55 years of age or older, and were capable of completing, or had access to assistance in completing, the SGBA-5 via a web-hosted survey (the URL was emailed to participants after they indicated to the research team that they were interested in participating). All participants in both arms provided written consent prior to participation in the study. The test-retest study was approved by and conducted in compliance with the regulations of the Ontario Tech University Research Ethics Board (REB # 17477).

### Procedure

Eligible participants completed the SGBA-5 twice, at least two weeks apart; they also completed a demographic questionnaire prior to completing the SGBA-5 for the first time. For the students, the SGBA-5 was completed on paper in-person; for the older adults, the SGBA-5 was completed online.

### Statistical analysis

The primary test statistics used to evaluate the test-retest reliability in each sample were Cohen’s kappa (κ) coefficient of agreement for the categorical sex variable and intraclass correlation coefficient of agreement (${ICC}_{\left(A,1\right)}$) for the four gendered aspect of health continuum variables at α=.05. The threshold for determining appropriate reliability of the SGBA-5 for use in research were set *a priori* as (κ≥.7) and $\left({ICC}_{\left(A,1\right)}\geq \mathrm{.7}\right)$ [18,42–45]. P-values are reported but were not used as a threshold to determine scale item reliability because the magnitude of the κ and ${ICC}_{\left(A,1\right)}$ coefficients (how similar a participant’s scores are between the test and retest) more directly assess a measurement tool’s reliability than calculating the probability that that tool’s results could be due to chance [18,44]. Secondary reliability analyses of the tool were conducted to quantify the standard error of measurement (SE_M_) for each of the four gendered aspect of health continuums, and sensitivity analysis were conducted using the sample’s demographic variables. SE_M_ results are presented as percentages of the feminine to masculine continuum used to quantify the four gendered aspects of health addressed in this tool. These SE_M_ percentages reflect the minimum difference (measured in % of the full continuum) between two observations of the same individual that would be needed to find a difference over time that is unlikely to be explained entirely by error [44]. Since this validity and reliability analysis is not assessing whether there are meaningful changes in participant’s experiences of these gendered aspects overtime, the SE_M_ percentages are instead presented to help demonstrate the typical amounts of variation that could be expected in an individual participant’s answers to the gendered aspect of health scale items. This allows for more confidence in identification of when there are meaningful differences between participants, i.e., if the difference between response of participant 1 and participant 2 is larger than the SE_M_ of that scale item, then we can suggest that there is likely a true difference between participants for that scale item.

All statistical analyses were conducted in the *R* statistical programming language (version 4.3.1: *Beagle Scouts*) along with the packages *tidyverse* (version 2.0.0), *irr* (version 0.84.1), *tableone* (version 0.13.2), and *ggpubr* (version 0.6.0) [46–50].

### Sample size

The target minimum sample size of 62 participants per arm completing the first (test) component of the study was derived by first plotting predicted minimum sample sizes using the Intraclass Transformation method and Optimal Design Approximation methods through a range of predicted reliability coefficients, confidence intervals, and minimum acceptable reliability coefficients [18,51,52]. Then, the more conservative estimate (n = 42), was identified for a predicted reliability coefficient of 0.85 (CI from 0.80 to 0.90). This number was rounded up to 50 to mitigate potential overestimation of the predicted reliability and then after accounting for a potential dropout rate of 25%, the target minimum sample size for the test-retest study was set as n = 62 [18].

## Results

### Initial design and development

The initial design process and validity testing culminated in the creation of the first full version of the SGBA-5. This version used five items (one biological sex item, four gendered aspect of health items) that had similar phrasings to those presented in Table 1 (which reports the phrasings used in version 1.0 of the SGBA-5. Version 1.0 of the SGBA-5 can be found in S1 File.). Small group feedback from health sciences researchers suggested that the SGBA-5 had good face validity for use in health sciences research where sex or gender are not the primary focus. The SGBA-5 was then presented to the Delphi experts to assess its content validity.

### Delphi expert consensus

**Fig 2:**
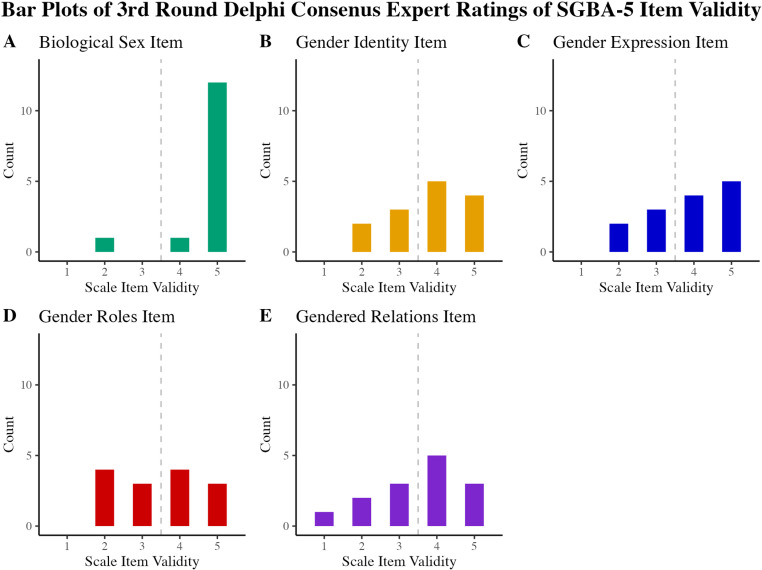
Frequency of 3rd round of Delphi consensus expert ratings of scale item validity.

Summary statistics from the Delphi study are presented in Table 2. The Delphi study was concluded after three rounds. The experts reached a consensus on all five items; only the categorical sex question met the *a priori* threshold for agreement of 75% of experts rating the item 4 or 5 out of 5 (93% agreement, median = 5.0, IQR = 0.0). Fig 2 is a stacked bar plot that shows the distribution of ratings for each scale item in the final round of the study. The optional feedback provided by the Delphi experts did not provide new insight into, or constructive critique, of the SGBA-5.

**Table 2:** Final round summary and test statistics from a Delphi consensus on the construct validity of the novel SGBA-5 tool for SGBA in health sciences research.

SGBA-5 Item	Delphi Round	Median (IQR)	% of Ratings ≥ 4 out of 5^a^	Between Round ΔCV^b^
Sex	3	5.0 (0.0)	**92.9**	N/A
Gender Identity	3	4.0 (1.8)	64.3	**0.000**
Gender Expression	3	4.0 (2.0)	64.3	**0.005**
Gender Roles	3	3.5 (1.8)	50.0	**0.069**
Gendered Relations	3	4.0 (1.0)	57.1	**0.053**

^a^Threshold for consensus agreement for an item’s validity was set a priori at $\begin{array}{c} \geq 75\mathrm{\backslash nonumber}\;\% \end{array}$.

^b^Threshold for consensus non-agreement for an item’s validity was set a priori at a ΔCV<0.15 between rounds.

### Test-retest study

Of the eligible student participants that completed the test round of the test-retest study (n = 102), 87% (n = 89) completed both rounds. Among older adults, 89 completed the first round of the test-retest study, and 80% (n = 71) completed both rounds. Table 3 displays the demographic characteristics for both the university student and older adult arms of the test-retest study.

**Table 3:** Demographic characteristics of test-retest participants by study arm.

Demographic Characteristic	Student Arm (n = 89)	Older Adult Arm (n = 71)
Preferred Language: (n (%))		
English	88 (98.9)	69 (97.2)
Age in years (mean, (SD))	20.9 (4.07)	66.1 (6.01)
Highest Level of Education: (n (%))		
High School	57 (64.0)	–
Some Post-Secondary/Trade Education	19 (21.3)	(*s*)
Completed Post-Secondary Degree/Diploma/Trade Certification	(*s*)	31 (43.7)
Professional/Graduate Degree or Higher	(*s*)	31 (43.7)
Prefer Not to Answer	–	(*s*)
Household Annual Income:		
$0 - $29,999 CAD	23 (25.8)	8 (11.3)
$30,000 - $59,999 CAD	12 (13.5)	17 (23.9)
$60,000 - $99,999 CAD	22 (24.7)	18 (25.4)
$100,000 CAD or more	32 (36.0)	19 (26.8)
Prefer Not to Answer	–	9 (12.7)
Ethnic/Cultural Origin^a^ (n (%))		
North American	26 (29.2)	41 (58.6)
South American	7 (7.9)	(*s*)
African	5 (5.6)	(*s*)
European	36 (40.4)	33 (47.1)
Middle Eastern	5 (5.6)	(*s*)
South Asian	16 (18.0)	(*s*)
East Asian	8 (9.0)	(*s*)
None of the Above	5 (5.6)	–

(*s*), cell sizes of < 5 were suppressed.

^a^Participants were able to select more than one option for their ethnic/cultural origin[s] therefore percentages are individual comparisons to the entire study population and do not sum to 100% across categories.

Results from the university students who completed both rounds of the test-retest study are presented in Table 4. There was complete agreement between test and retest answers for the categorical sex item $\begin{array}{c} (\kappa =1.00,95\\ \%CI(1.00,1.00)) \end{array}$. All four gendered aspects of health had ${ICC}_{\left(A,1\right)}$ scores greater than the *a priori* threshold of ${ICC}_{\left(A,1\right)}\geq \mathrm{.7}$, ranging from the gender roles item which had an $\begin{array}{c} {ICC}_{\left(A,1\right)}=\mathrm{.899},95\\ \%CI(\mathrm{.851},\mathrm{.933}) \end{array}$ to a high of $\begin{array}{c} {ICC}_{\left(A,1\right)}=\mathrm{.899},95\\ \%CI(\mathrm{.851},\mathrm{.933}) \end{array}$ for the gender identity item.

**Table 4:** SGBA-5 test-retest reliability results: Student arm.

Scale Item	Cohen’s κ^a^	${ICC}_{\left(A,1\right)}$ ^a^	95% CI	p-value	SE_M_ (%)
*Sex*	**1.00**	–	(1.00, 1.00)	–	–
*Gender Identity*	–	**0.966**	(0.942, 0.979)	<.0001	9.2
*Gender Expression*	–	**0.948**	(0.919, 0.966)	<.0001	11.8
*Gender Roles*	–	**0.899**	(0.851, 0.933)	<.0001	16.3
*Gendered Relations*	–	**0.955**	(0.932, 0.970)	<.0001	10.6

^a^Threshold for significance was set *a priori* as Cohen’s κ or ${ICC}_{\left(A,1\right)}\geq \mathrm{.7}$.

Sensitivity analyses of the student arm’s gendered aspects of health ${ICC}_{\left(A,1\right)}$ coefficients showed no significant differences when grouped by demographic characteristics, and all subgroup ${ICC}_{\left(A,1\right)}$ coefficients were greater than the 0.7 *a priori* threshold. More detailed results for each sensitivity analysis are presented in the S1 Table.

Results from the older adult arm of the test-retest study are presented in Table 5. There was perfect agreement between test and retest answers for the categorical sex item $\begin{array}{c} (\kappa =1.00,95\\ \%CI(1.00,1.00)) \end{array}$. All four gendered aspects of health had ${ICC}_{\left(A,1\right)}$ scores greater than the *a priori* threshold of ${ICC}_{\left(A,1\right)}\geq \mathrm{.7}$, which ranged from the gender roles item which had an $\begin{array}{c} {ICC}_{\left(A,1\right)}=\mathrm{.865},95\\ \%CI(\mathrm{.772},\mathrm{.920}) \end{array}$ to a high of $\begin{array}{c} {ICC}_{\left(A,1\right)}=\mathrm{.944},95\\ \%CI(\mathrm{.909},\mathrm{.965}) \end{array}$ for the gendered relations item. Sensitivity analyses of the older adult arm of the test-retest study stratified by self-reported demographic characteristics had all sub-groups scoring higher than the *a priori* threshold of ${ICC}_{\left(A,1\right)}\geq \mathrm{.7}$, which are detailed in the S2 Table.

**Table 5:** SGBA-5 test-retest reliability results: Older adult arm.

Scale Item	Cohen’s κ^a^	${ICC}_{\left(A,1\right)}$ ^a^	95% CI	p-value	SE_M_ (%)
*Sex*	**1.00**	–	(1.00, 1.00)	–	–
*Gender Identity*	–	**0.927**	(0.886, 0.954)	<.0001	13.8
*Gender Expression*	–	**0.899**	(0.840, 0.937)	<.0001	15.8
*Gender Roles*	–	**0.865**	(0.772, 0.920)	<.0001	17.8
*Gendered Relations*	–	**0.944**	(0.909, 0.965)	<.0001	11.6

^a^Threshold for significance was set *a priori* as Cohen’s κ or ${ICC}_{\left(A,1\right)}\geq \mathrm{.7}$.

## Discussion

The purpose of the work presented in this paper was to conduct initial assessment of a novel tool for SGBA. The tool was designed to provide more meaningful insight into the variation and multidimensionality of sex and gender than what is possible using categorical option tools, without also significantly increasing the time commitment and workload for the researchers who use it. A strength of the proposed SGBA-5 tool is that the responses of gender-diverse persons can be included and analyzed without necessitating the large sample sizes (often an n of 500 or more assuming a 1% proportion) that are required to analyze small-proportion nominal groups.

This paper reports the first formal validity and reliability testing of the SGBA-5. Specifically, our testing was designed to determine whether the SGBA-5 was robust enough to recommend further assessment and trial use in health sciences research studies in which sex or gender are not primary variables. The Delphi expert consensus study on the SGBA-5’s validity provided strong support for the biological sex item. While the gendered aspect of health items did not meet the conservative *a priori* threshold for consensus agreement (75%), the items were not rejected either. The test-retest study of the SGBA-5’s reliability demonstrated strong reliability for all items in both population arms, which was further bolstered by the reliability coefficients robustness in secondary analysis, and narrow SE_M_ margins. To our knowledge, this is the first SGBA tool that has been created with the specific intention of enabling researchers to integrate SGBA into health sciences research when sex or gender are not primary variables of interest.

The Delphi expert consensus study of health researchers from across Canada rated the validity of the SGBA-5’s biological sex item as being highly valid $\begin{array}{c} (>90\\ \%rated\geq 4of5) \end{array}$, which is consistent with what we expected since the use of a nominal-categorical item to report biological sex is already the most commonly used and accepted way of incorporating biological sex into health research outside of specialized areas of sex-based research [2,6,8,11,12]. The Delphi expert’s ratings of the gendered aspects of health items that used a feminine-masculine continuum did not meet our intentionally conservative *a priori* consensus target of at least 75% of validity ratings being 4 or 5 out of 5, but all of the items had median ratings of > 3 out of 5 with at least 50% of ratings being 4 or 5 out of 5, both of which are thresholds that have been used to define consensus in Delphi expert consensus studies [18,40,41,53–56]. This suggests that while experts were not unanimous in their endorsement of the use the gendered aspects of health items utilizing a feminine-masculine continuum, they did not view any of the items as invalid either. This mixed response from the Delphi experts may be reflective of the broader inconsistencies in health researchers’ definitions of sex and gender in a health science context. As Haverfield and Tannenbaum noted in their evaluation of nearly 40,000 grant applications, both researchers and grant evaluators had difficulty in consistently applying the principles of SGBA despite having to complete mandatory education modules on the topic [10]. It is perhaps unsurprising that the health researchers in our study, whose primary focus is not sex or gender research, were hesitant to give a higher validity rating to scale items that operationalize gendered aspects of health. It is also possible that some of the Delphi expert’s validity ratings may have been negatively influenced by misunderstanding of the purpose, use cases, or scope of the SGBA-5, particularly given the current diversity of understandings and conceptualizations of gender [2,6,8,10,26,57–60].

The test-retest reliability study demonstrated strong reliability among all SGBA-5 items and across both study arms. The student and older adult arms had perfect test-retest reliability for the biological sex item (κ=1.00), and the reliability coefficients for the gendered aspect of health continuum items were all well above the *a priori* acceptable reliability threshold of ${ICC}_{\left(A,1\right)}\geq \mathrm{.7}$ at an α=.05. Both the student and older adult samples were relatively homogeneous, and thus should not be generalized to all university students or all older adults; however, the reliability results for all the gendered aspect of health items were further supported by all the sub-group coefficients in the demographic-based sensitivity analyses surpassing the *a priori* reliability coefficient threshold as well. Additionally, calculated SE_M_ percentages from the test-retest data (ranging from 9.2% for gender identity item in the student arm to 17.8% for the older adult gender roles item) support the supposition that an analogue continuum measure can provide more precise and detailed information on the variation that occurs within gendered aspects of health than a nominal or most ordinal measures could.

The SGBA-5 can allow health sciences researchers to incorporate SGBA more easily into their study designs where sex and gender are not the primary focus. The SGBA-5 is designed to be easily added into existing demographic questionnaires or other pre-study procedures already used by researchers. The tool on paper takes up a maximum of one page and takes up a similar amount of space when administered digitally. When combined with the SGBA-5’s quick time-to-completion (1–2 minutes) it is our hope that the SGBA-5 represents a more implementable option for researchers who want to be incorporating more detailed SGBA into their work. The SGBA-5 provides researchers with a way to measure sex and gender separately beyond the oft used categorical sex and gender tick box options (which are unlikely to be differentiable unless the study’s sample size is very large). The SGBA-5 can provide researchers with descriptive insights into their sample (such as whether the sample has an uneven distribution across one or more of the gendered aspects of health) as well as allowing for the assessment of potential confounding occurring between biological sex, the four measured gendered aspects of health, and the study’s primary outcome[s] of interest. A more detailed example data analysis using simulated SGBA-5 and outcome measures can be found at https://github.com/putman-a/SGBA-5_example_analysis [38].

The strengths of the validity assessment of the SGBA-5 include the breadth of researcher insights in the Delphi validity from recruiting experts from institutions across Canada, and the more conservative thresholds for consensus used when assessing the SGBA-5’s validity. The strengths of the test-retest reliability assessment include having conducted test-retest with two separate populations, using two different mediums, and having conducted sensitivity analyses of the reliability coefficients across sample demographics. A potential limitation of the Delphi expert consensus study was the method of expert selection. The research team attempted to mitigate self-selection bias from the experts by contacting health sciences Deans of Canadian institutions and requesting that they nominate a researcher to participate, but this type of sampling does not necessarily generate a representative sample of the entire population of Canadian health researchers who could have provided expertise for this Delphi study. Further, this Delphi study’s results showed that the experts’ assessments of the SGBA’s validity quickly reached stability between rounds (all ΔCV<.07 between the 2^nd^ and 3^rd^ rounds), which may suggest that a larger quantity of experts or experts with more diverse opinions could provide more insight into the SGBA-5’s validity. Additionally, while the test-retest study being conducted with two populations is a strength, it is important to caution against overgeneralization of these results as the nature of the online survey given to the older adult sample only includes those who could comfortably complete the questionnaire on an internet enabled device, and the university student population was recruited from just two programs (Kinesiology and Psychology) which may not be representative of Canadian university students in general. Further evaluation of the SGBA-5 should investigate the different aspects of scale validity and reliability across diverse population groups.

## Conclusion

To our knowledge, this paper marks the first validity and reliability testing of a novel tool designed for implementation of SGBA in health sciences research where sex or gender are not primary variables. We found that all five items in the SGBA-5 were reliable, that the biological sex item was deemed valid by the Delphi expert panel, and that the measurement of the gendered aspects of health items using a feminine-masculine continuum may be valid for use in health sciences research. Our testing of the SGBA-5 has shown promise for further development and applications of the SGBA-5.

## Supporting information

S1 File Sex- and gender-based analysis tool 5-item (v1.0). (PDF)

S1 Table Sensitivity analyses of gendered aspects of health test-retest reliability coefficients: Student arm. (DOCX)

S2 Table Sensitivity analyses of gendered aspects of health test-retest reliability coefficients: Older adult arm. (DOCX)
