# Supplementary material for: Initial validity and reliability testing of the SGBA-5
Source: PLoS One. 2025 May 16;20(5):e0323834. doi: 10.1371/journal.pone.0323834 (PMC12084046; doi:10.1371/journal.pone.0323834)
Supplement: S1 File — (PDF) [file pone.0323834.s001.pdf]

v1.0

Sex- and Gender-Based Analysis Tool – 5 Item

Putman & Dogra

TABLE OF CONTENTS

SGBA-5 Researcher Instructions 2
Steps for Using Tool 2
SGBA-5 Tool Implementation 3
Researcher Notes 3
Paper-Based Use 3
Computerized/Digital Use 3
Sex- and Gender-Based Analysis Tool – 5 Item (SGBA-5) 4
Context & Background of SGBA-5 5
Rationale for Tool Creation 5
Example Use Cases 6
Example Appropriate SGBA-5 Use Cases. 6
Example INAPPROPRIATE Use Cases for the SGBA-5 6
FAQ 7
References 9

Sex- and Gender-Based Analysis Tool – 5 Item © 2024 by Putman & Dogra is licensed under **CC BY 4.0**. To view a copy of this license, visit http://creativecommons.org/licenses/by/4.0/

SGBA-5 RESEARCHER INSTRUCTIONS

This instruction page is intended for the researchers and professionals who will use the Sex- and Gender-Based Analysis tool – 5 item (SGBA-5), NOT for the participants completing the tool.

The SGBA-5 allows the participants in your study to indicate their ***biological sex*** at birth using a categorical tick-box question, and to report their self-perceived relations to 4 ***gendered aspects of health*** on a feminine to masculine continuum using an analogue continuum measure.

Before beginning your study, consider **when** you plan on asking your participants to complete the tool. Doing so can help you minimize potential priming effects that the tool might have on the measures that are the primary focus of your study. For example, this tool could be completed as part of a pre-study demographic questionnaire or could be administered after your intervention has concluded.

Steps for Using Tool

1) Please give the tool to your participants to fill in without further instruction. The SGBA-5 can be integrated as part of a larger questionnaire in your study.
2) Please *do not* define the terms 'masculine' or 'feminine' for the participants if asked. It is important that the participants complete the SGBA-5 based on *their own understanding* of these terms.
   a) If participants are not sure or are uncomfortable with responding to an item, please allow them to skip the item [record as 'prefer not to answer' in your data] or provide their best guess.
   b) If a participant is concerned that their response to the gendered aspect of health question differs depending on the situation, that is not a problem. Their responses on the analogue scales should reflect their 'typical experience' – which will often mean that the participant needs to average-out or weigh their response.
3) If the tool was completed on a computer, you can skip this step. If the tool was completed on paper:
   a) Responses from the biological sex item can be directly coded into your data set for analysis.
   b) Recording responses from the measures of gendered aspects of health will require use of a ruler to measure **in mm** the distance of the tick for each question from the one of the feminine or masculine anchors. *The chosen anchor needs to be consistent for all measurements within the same study*.
      i) Please note, the 4 feminine to masculine lines are exactly 100mm.
      ii) It is best practice to measure each marker on their respective lines at least twice to help limit measurement error.

Sex- and Gender-Based Analysis Tool – 5 Item © 2024 by Putman & Dogra is licensed under **CC BY 4.0**. To view a copy of this license, visit http://creativecommons.org/licenses/by/4.0/

SGBA-5 Tool Implementation

Researcher Notes

The SGBA-5 is designed for with-in study sex-and gender-based analyses (SGBA) in studies where ***sex or gender are not primary variables*** in the research question being studied. The scale ***has not been validated for use specifically with sex and gender minority populations*** [intersex persons, non-binary persons, etc.], however, that does not preclude members of those minority populations from completing the SGBA-5 as a part of a research study recruiting from the general population.

The intent of the gendered aspects of health items in the SGBA-5 are to provide a continuous metric that assess different aspects of gender that affect health in a way that is meaningful to your study sample. Each of these items reflect a different [but nonexclusive] path through which gender can impact health, thus the gendered aspect ***items should not be combined into a singular score***. The SGBA-5 is not meant for use as a gender classification tool and is not meant to be used to describe any individual participant.

Paper-Based Use

Please print [or copy and paste into your demographic questionnaire] the print version of the SGBA-5 that is included the following page. **Do not alter the size, font, or wording**.

Computerized/Digital Use

Using your survey software of choice, the following criteria must be met:

- Item names, instructions, headings, and descriptions are to be **copied exactly** from the print version [which can be found on the following page].
- The biological sex item is coded as a single-choice categorical option of ‘Female’, ‘Male’, or ‘Intersex’.
- The gendered aspect of health items must be coded as continuous sliders/analogue scales that include a **range of exactly 101 possible responses**, [0 – 100], or [1 – 101] are the two most likely response option parameters you will encounter, but this differs by software.
- Like the print version, the sliders/continuums used need to be anchored with the left side being labelled as ‘feminine’ and the right side being labelled as ‘masculine’ and the **numerical values of the anchors should not be visible**.
- The slider should **not have their numerical response be visible** to the participant at any point.
- The responses of the sliders/scales are to be **recorded and exported as is** [integer values with a 101-point range]. The responses should not be averaged, totaled, or otherwise transformed by the survey software prior to export for data analysis.
- If the survey software has a “slider button” or similar which the user moves to select their response and it appears to the user prior to their response, have the button begin at the middle of the scale rather than at one of the anchors.

Sex- and Gender-Based Analysis Tool – 5 Item © 2024 by Putman & Dogra is licensed under **CC BY 4.0**. To view a copy of this license, visit http://creativecommons.org/licenses/by/4.0/

SEX- AND GENDER-BASED ANALYSIS TOOL – 5 ITEM (SGBA-5)

version 1.0

Sex - Please indicate your sex assigned at birth:

- ☐ Female
- ☐ Male
- ☐ Intersex

Gender – For the following 4 items, please place a mark on the corresponding lines.

***Identity* -** How would you generally describe yourself on a feminine to masculine continuum?

feminine ———————————————————— masculine

***Expression* -** How would you describe your typical behaviour and mannerisms on a feminine to masculine continuum?

feminine ———————————————————— masculine

***Roles* -** How would you describe your day-to-day responsibilities on a feminine to masculine continuum?

feminine ———————————————————— masculine

***Relations* -** How would you generally describe how your friends, colleagues, and authority figures in your life typically treat you on a feminine to masculine continuum?

feminine ———————————————————— masculine

Sex- and Gender-Based Analysis Tool – 5 Item © 2024 by Putman & Dogra is licensed under **CC BY 4.0**. To view a copy of this license, visit http://creativecommons.org/licenses/by/4.0/

Context & Background of SGBA-5

Rationale for Tool Creation

Despite the support and efforts of the Tri-Council Agencies, sex- and gender-based analysis (SGBA) is under-utilized and often poorly implemented in health research outside of research where sex or gender are primary variables of interest. The CIHR has noted that even researchers and grant reviewers who receive training on SGBA cannot reliably differentiate between sex and gender.[1]

Importantly, the most prevalent SGBA tools available for use in health research either rely on nominal categorization of male/female or man/woman/other[sic] or require lengthy questionnaires which can be impractical to implement and are rarely, if ever, used outside of sex- and gender-focused research.

Given this, the aim for the measurement tool that became the SGBA-5 was to create a short, easy to implement tool that enables researchers to incorporate more nuanced SGBA into studies where sex and gender are not the primary focus. There is evidence to support that the factors incorporated into this tool have an impact on health outcomes, more detailed explanations of which can be found in the thesis work *Initial Validity and Reliability Testing of the SGBA*-5 by Putman and in the validity and reliability paper by Putman, Cole, & Dogra. The authors do not claim that these factors are an exhaustive list nor that any sort of averaging/summing/other mathematical manipulation will result in a holistic measure of gender.

We accept that this tool is not perfect and hope that future work on the SGBA-5 will lead to updates beyond this 1.0 version. We believe that this tool has the potential to be one of many such SGBA implementation tools available to researchers, and hope that this sparks further work in the area.

Sex- and Gender-Based Analysis Tool – 5 Item © 2024 by Putman & Dogra is licensed under **CC BY 4.0**. To view a copy of this license, visit http://creativecommons.org/licenses/by/4.0/

EXAMPLE USE CASES

A more extensive example analysis, including interpretation guides and the code used to conduct that analysis can be found at the following url: https://github.com/putman-a/SGBA-5_example_analysis

Example Appropriate SGBA-5 Use Cases.

- Studies where sex or gender are not primary variables of interest.
- Studies with samples that draw from populations with relatively homogenous sociodemographic characteristics [i.e., university students, a workplace/office, a sports team, etc.].
- Small-to-medium clinical interventions.

Example INAPPROPRIATE Use Cases for the SGBA-5

- Work exclusively focused on sex- and/or gender minority populations.
- Research where sex or gender are a primary variable of interest.
- Studies with samples which intentionally utilize highly heterogeneous samples.
  - Though if a study were looking at comparing samples that are distinguished by location/year/etc., the SGBA-5 would be appropriate for use in within-sample analyses of each relatively homogenous sample, but not for generalization between distinct samples.
- Studies which span multiple years.
  - The SGBA-5 has not been validated for this use case; further research is needed to see whether the SGBA-5's findings remain valid over time long periods of time [without follow-up measurements]. In the meantime, caution is suggested.

Sex- and Gender-Based Analysis Tool – 5 Item © 2024 by Putman & Dogra is licensed under **CC BY 4.0**. To view a copy of this license, visit http://creativecommons.org/licenses/by/4.0/

FAQ

Can the values from the SGBA-5's gendered aspects of health items be generalized?

No.

One of the key methodological goals in designing the SGBA-5 is to allow for **participant self-definition** of femininity and masculinity as it relates to their lived experience. Implementing this judiciously required the restriction of generalization of the SGBA-5's results and means that **the SGBA-5 measures relative differences between participants**, not a characteristic of the participant that exists outside of the context of their lived experiences. Since most health research is conducted with relatively homogenous populations (i.e., participants live in a concentrated geographic area, are of similar age, SES, etc.) those self-definitions should be comparable within those known parameters but should not be considered to have meaning outside of that specific context or sample.

Why gender identity, expression, roles, and relations? Why not ______?

The selection of these specific gendered factors were informed by gender and health frameworks produced by research organizations such as the Women's Health Research Network[2] and the Centre for Gender and Sexual Health Equity.[3] We then reviewed health science literature for evidence of these factors impacting health. For example, valuing modern stereotypical western-masculine gender expression of personality traits [individualism, toughness, bravery, etc.] has been repeatedly associated with experiencing more severe symptoms and longer recovery periods among those who develop post-traumatic stress disorder.[4–6]

Aren't masculinity and femininity separate things?

The SGBA-5 does not measure the entirety of gender, it is not comprehensive of all aspects of gender nor is it representative of all conceptualizations of gender. **The SGBA-5 is only designed to assess how certain gendered factors can influence health along a feminine-masculine continuum**, which itself was derived from current health literature.

There has been a sizable amount of health literature that assesses masculinity and femininity as separate personality constructs[7–11] [i.e., the Bem scale and its derivates, Androgyny theory etc.]. However, the authors are **not aware of any health research that shows that utilizing masculinity and femininity as distinct constructs is valid or useful when assessing aspects of gender which are not solely personality-based** [such as identity, expectations/roles, etc.]. We do not claim to have novel insight into how to classify or study gender as a construct or grouping of constructs. Rather, the SGBA-5 was created to be a short and easy to implement tool measuring within-sample variation of biological sex and four of the aspects of gender which have been shown to impact the health.

Sex- and Gender-Based Analysis Tool – 5 Item © 2024 by Putman & Dogra is licensed under **CC BY 4.0**. To view a copy of this license, visit http://creativecommons.org/licenses/by/4.0/

What about intersex/genderqueer/non-binary/2 spirit people?

Using current methods for SGBA in health research studies on a general population, people who are a part of gender or sexual minorities cannot be included in the typical disaggregated analysis due to privacy and identifiability concerns. [e.g., if a study had 50 participants and 2 of them are trans/non-binary, any analysis of those trans/non-binary participants cannot be ethically published due to the privacy and confidentiality concerns of small cell sizes]. **The SGBA-5 asks participants to record their gendered aspect of health responses on a feminine to masculine continuum and does not claim to measure anything beyond that.**

A part of the trade-off for conciseness in this tool is the detail and scope of it. Therefore, the **SGBA-5 was not designed to be comprehensive enough for work specifically with sex and gender minority populations**. However, that does not mean that the SGBA-5 cannot be completed by people in those minority populations, as they too exist in, and are influenced by, the cultural and contextual impactions of these gendered aspects of health. We do not endorse using this tool for research that is specific to sex and gender minority populations.

Sex- and Gender-Based Analysis Tool – 5 Item © 2024 by Putman & Dogra is licensed under **CC BY 4.0**. To view a copy of this license, visit http://creativecommons.org/licenses/by/4.0/

REFERENCES

1. Haverfield J, Tannenbaum C. A 10-year longitudinal evaluation of science policy interventions to promote sex and gender in health research. *Health Research Policy and Systems*. 2021;19(1):1-12. doi:10.1186/s12961-021-00741-x

2. Johnson J, Greaves L, Repta R. Better Science with Sex and Gender: A Primer for Health Research. Published online 2007. https://cewh.ca/wp-content/uploads/2012/05/2007_BetterSciencewithSexandGenderPrimerforHealthResearch.pdf

3. Lowik A, Cameron J, Dame J, et al. *Tool #1: Determining & Communicating Eligibility*. University of British Columbia; 2022. Accessed November 11, 2022. https://cgshe.ca/app/uploads/2022/01/GSMM-Research-Equity-Tool-1.pdf

4. Christiansen DM, Berke ET. Gender- and Sex-Based Contributors to Sex Differences in PTSD. *Curr Psychiatry Rep*. 2020;22(4):19. doi:10.1007/s11920-020-1140-y

5. Morrison JA. Masculinity moderates the relationship between symptoms of PTSD and cardiac-related health behaviors in male veterans. *Psychology of Men & Masculinity*. 2012;13:158-165. doi:10.1037/a0024186

6. Caddick N, Smith B, Phoenix C. Male combat veterans’ narratives of PTSD, masculinity, and health. *Sociology of Health & Illness*. 2015;37(1):97-111. doi:10.1111/1467-9566.12183

7. Auster CJ. Bem Sex-Role Inventory. In: Zeigler-Hill V, Shackelford TK, eds. *Encyclopedia of Personality and Individual Differences*. Springer International Publishing; 2020:445-449. doi:10.1007/978-3-319-24612-3_1207

8. Bem SL. The measurement of psychological androgyny. *J Consult Clin Psychol*. 1974;42(2):155-162.

9. Carver LF, Vafaei A, Guerra R, Freire A, Phillips SP. Gender Differences: Examination of the 12-Item Bem Sex Role Inventory (BSRI-12) in an Older Brazilian Population. *PLOS ONE*. 2013;8(10):e76356. doi:10.1371/journal.pone.0076356

10. Choi N, Fuqua DR, Newman JL. The Bem Sex-Role Inventory: Continuing Theoretical Problems. *Educational and Psychological Measurement*. 2008;68(5):881-900. doi:10.1177/0013164408315267

11. Davis S. Bem Sex-Role Inventory | Definition, Scoring, & Uses | Britannica. In: *Encyclopedia Britannica*.; 2017. Accessed January 23, 2023. https://www.britannica.com/science/Bem-Sex-Role-Inventory

Sex- and Gender-Based Analysis Tool – 5 Item © 2024 by Putman & Dogra is licensed under **CC BY 4.0**. To view a copy of this license, visit http://creativecommons.org/licenses/by/4.0/
