# Supplementary material for: Initial validity and reliability testing of the SGBA-5
Source: PLoS One. 2025 May 16;20(5):e0323834. doi: 10.1371/journal.pone.0323834 (PMC12084046; doi:10.1371/journal.pone.0323834)
Supplement: S1 Table — (DOCX) [file pone.0323834.s002.docx]

S1 Table. Sensitivity analyses of gendered aspects of health test-retest reliability coefficients: Student arm.

| Sensitivity Variable | Scale Item | ICC | 95%CI | p-value |
| --- | --- | --- | --- | --- |
| Education: |  |  |  |  |
| High School | Gender Identity | **0.956** | (0.921, 0.975) | < .0001 |
| Some Post Sec. | Gender Identity | **0.987** | (0.966, 0.995) | < .0001 |
| All Other | Gender Identity | **0.957** | (0.848, 0.987) | < .0001 |
| High School | Gender Expressions | **0.948** | (0.914, 0.969) | < .0001 |
| Some Post Sec. | Gender Expressions | **0.957** | (0.848, 0.985) | < .0001 |
| All Other | Gender Expressions | **0.934** | (0.804, 0.979) | < .0001 |
| High School | Gender Role | **0.857** | (0.77, 0.913) | < .0001 |
| Some Post Sec. | Gender Role | **0.972** | (0.928, 0.989) | < .0001 |
| All Other | Gender Role | **0.928** | (0.783, 0.977) | < .0001 |
| High School | Gender Relations | **0.942** | (0.903, 0.965) | < .0001 |
| Some Post Sec. | Gender Relations | **0.964** | (0.911, 0.986) | < .0001 |
| All Other | Gender Relations | **0.984** | (0.947, 0.995) | < .0001 |
| Annual Household Income: |  |  |  |  |
| $0 - $29,999 CAD | Gender Identity | **0.980** | (0.951, 0.992) | < .0001 |
| $30,000 - $59,999 CAD | Gender Identity | **0.925** | (0.755, 0.978) | < .0001 |
| $60,000 - $99,999 CAD | Gender Identity | **0.970** | (0.929, 0.987) | < .0001 |
| $100,000 CAD or more | Gender Identity | **0.959** | (0.904, 0.982) | < .0001 |
| $0 - $29,999 CAD | Gender Expressions | **0.974** | (0.941, 0.989) | < .0001 |
| $30,000 - $59,999 CAD | Gender Expressions | **0.951** | (0.792, 0.987) | < .0001 |
| $60,000 - $99,999 CAD | Gender Expressions | **0.945** | (0.872, 0.977) | < .0001 |
| $100,000 CAD or more | Gender Expressions | **0.930** | (0.852, 0.966) | < .0001 |
| $0 - $29,999 CAD | Gender Role | **0.929** | (0.841, 0.969) | < .0001 |
| $30,000 - $59,999 CAD | Gender Role | **0.931** | (0.787, 0.979) | < .0001 |
| $60,000 - $99,999 CAD | Gender Role | **0.854** | (0.682, 0.936) | < .0001 |
| $100,000 CAD or more | Gender Role | **0.896** | (0.800, 0.948) | < .0001 |
| $0 - $29,999 CAD | Gender Relations | **0.968** | (0.925, 0.986) | < .0001 |
| $30,000 - $59,999 CAD | Gender Relations | **0.974** | (0.915, 0.992) | < .0001 |
| $60,000 - $99,999 CAD | Gender Relations | **0.954** | (0.895, 0.981) | < .0001 |
| $100,000 CAD or more | Gender Relations | **0.936** | (0.871, 0.968) | < .0001 |
| Cultural / Ethnic Origin: |  |  |  |  |
| Americas | Gender Identity | **0.965** | (0.930, 0.983) | < .0001 |
| Europe | Gender Identity | **0.963** | (0.925, 0.982) | < .0001 |
| All Other | Gender Identity | **0.964** | (0.926, 0.982) | < .0001 |
| Americas | Gender Expressions | **0.926** | (0.854, 0.963) | < .0001 |
| Europe | Gender Expressions | **0.950** | (0.897, 0.975) | < .0001 |
| All Other | Gender Expressions | **0.957** | (0.918, 0.977) | < .0001 |
| Americas | Gender Role | **0.899** | (0.803, 0.949) | < .0001 |
| Europe | Gender Role | **0.938** | (0.882, 0.968) | < .0001 |
| All Other | Gender Role | **0.875** | (0.773, 0.933) | < .0001 |
| Americas | Gender Relations | **0.955** | (0.910, 0.977) | < .0001 |
| Europe | Gender Relations | **0.951** | (0.906, 0.975) | < .0001 |
| All Other | Gender Relations | **0.950** | (0.907, 0.974) | < .0001 |

**Note:** ICC_(A,1)_ were conducted for subgroupings which contained > 9 observations.
