# Supplementary material for: Initial validity and reliability testing of the SGBA-5
Source: PLoS One. 2025 May 16;20(5):e0323834. doi: 10.1371/journal.pone.0323834 (PMC12084046; doi:10.1371/journal.pone.0323834)
Supplement: S2 Table — (DOCX) [file pone.0323834.s003.docx]

S2 Table. Sensitivity analyses of gendered aspects of health test-retest reliability coefficients: Older adult arm.

| Sensitivity Variable | Scale Item | ICC | 95%CI | p-value |
| --- | --- | --- | --- | --- |
| Education |  |  |  |  |
| Secondary School | Gender Identity | **0.935** | (0.869, 0.968) | < .0001 |
| Professional | Gender Identity | **0.898** | (0.794, 0.950) | < .0001 |
| Secondary School | Gender Expressions | **0.940** | (0.875, 0.972) | < .0001 |
| Professional | Gender Expressions | **0.828** | (0.669, 0.914) | < .0001 |
| Secondary School | Gender Role | **0.888** | (0.778, 0.946) | < .0001 |
| Professional | Gender Role | **0.784** | (0.540, 0.904) | < .0001 |
| Secondary School | Gender Relations | **0.961** | (0.920, 0.981) | < .0001 |
| Professional | Gender Relations | **0.907** | (0.806, 0.957) | < .0001 |
| Household Income: |  |  |  |  |
| $30,000 - $59,999 CAD | Gender Identity | **0.919** | (0.754, 0.971) | < .0001 |
| $60,000 - $99,999 CAD | Gender Identity | **0.955** | (0.884, 0.983) | < .0001 |
| $100,000 CAD or more | Gender Identity | **0.860** | (0.677, 0.943) | < .0001 |
| $30,000 - $59,999 CAD | Gender Expressions | **0.747** | (0.410, 0.904) | .0003 |
| $60,000 - $99,999 CAD | Gender Expressions | **0.954** | (0.880, 0.983) | < .0001 |
| $100,000 CAD or more | Gender Expressions | **0.954** | (0.882, 0.983) | < .0001 |
| $30,000 - $59,999 CAD | Gender Role | **0.942** | (0.814, 0.983) | < .0001 |
| $60,000 - $99,999 CAD | Gender Role | **0.86** | (0.624, 0.949) | < .0001 |
| $100,000 CAD or more | Gender Role | **0.881** | (0.701, 0.956) | < .0001 |
| $30,000 - $59,999 CAD | Gender Relations | **0.93** | (0.817, 0.974) | < .0001 |
| $60,000 - $99,999 CAD | Gender Relations | **0.951** | (0.873, 0.982) | < .0001 |
| $100,000 CAD or more | Gender Relations | **0.933** | (0.820, 0.976) | < .0001 |
| Cultural / Ethnic Origin: |  |  |  |  |
| Americas | Gender Identity | **0.943** | (0.894, 0.969) | < .0001 |
| Europe | Gender Identity | **0.912** | (0.830, 0.956) | < .0001 |
| Americas | Gender Expressions | **0.935** | (0.880, 0.965) | < .0001 |
| Europe | Gender Expressions | **0.849** | (0.713, 0.924) | < .0001 |
| Americas | Gender Role | **0.895** | (0.792, 0.947) | < .0001 |
| Europe | Gender Role | **0.824** | (0.657, 0.915) | < .0001 |
| Americas | Gender Relations | **0.950** | (0.905, 0.974) | < .0001 |
| Europe | Gender Relations | **0.939** | (0.876, 0.970) | < .0001 |

**Note:** ICC_(A,1)_ were conducted for subgroupings which contained > 9 observations.
