# Supplementary material for: Initial validity and reliability testing of the SGBA-5
Source: PLoS One. 2025 May 16;20(5):e0323834. doi: 10.1371/journal.pone.0323834 (PMC12084046; doi:10.1371/journal.pone.0323834)
Supplement: Attachment — Submitted filename: Reviewer comments_19_01_2025.pdf [file pone.0323834.s007.pdf]

Thank you for your thoughtful revisions and responses to the previous round of comments. The manuscript reflects substantial effort in addressing key concerns, particularly in acknowledging the limitations of the sample diversity, providing insights into the reliability testing, and justifying the inclusion of the gendered constructs. These updates strengthen the study's foundational contribution as a preliminary step in the development and validation of the SGBA-5 tool. However, some areas still require further clarification and refinement to ensure the manuscript meets its full potential. Below are the comments and suggestions for further improvements.

General Decision: Resubmit with Minor Revisions

While the manuscript shows promise and significant progress has been made, a few remaining issues need to be addressed for clarity and to strengthen the theoretical and methodological underpinnings. The revisions required are minor and aimed at enhancing the transparency and robustness of the study.

Reviewer Comments

1. Delphi Study and Consensus

- The decision to halt the Delphi process after three rounds and include gendered items despite not meeting the 75% consensus threshold is partially justified. However, the rationale could be more robust. Specifically:
    - Provide a more detailed analysis of the expert disagreements and clarify whether the non-consensus stemmed from theoretical differences or practical concerns.
    - If feasible, summarize the key optional feedback received (even if it was not constructive) to illustrate the range of expert perspectives.

2. Sample Diversity

- While the manuscript acknowledges the homogeneity of the Delphi panel and test-retest populations, the implications of this limitation should be emphasized more strongly.
    - For example, discuss how the findings might differ in gender-diverse or underrepresented populations and suggest specific future research directions to address this gap.

3. Theoretical Framework

- The four gender constructs (identity, expression, roles, relations) are an important aspect of the SGBA-5, yet their theoretical justification could be expanded.
    - Include a brief discussion in the main text explaining why these constructs were chosen and how they influence health outcomes. While the reference to the thesis is helpful, a concise summary would provide more immediate context for readers.

4. Terminology

- The consistent use of "biological sex" aligns with Canadian research standards; however, complementing this with "sex assigned at birth" where appropriate would enhance inclusivity and align with best practices in gender-affirming contexts.

5. Clarity and Style

- While the manuscript is generally well-written, certain sections (e.g., discussion and methodology) would benefit from additional clarity. Simplify complex sentences and ensure consistent terminology throughout the text.
    - A final proofreading pass is recommended to ensure grammatical accuracy and improve readability.

6. Methodology Transparency

- The integration of feedback from the Delphi study could be described in greater detail.
    - For example, specify how disagreements or diverse opinions were handled in the revision process, even if optional feedback provided little constructive insight.

7. Supplementary Materials

- Key details from supplementary materials, such as the example analysis and methodological rationale, should be summarized briefly in the main text to make the paper more self-contained.

Conclusion

This manuscript represents an important step in advancing tools for SGBA in health research. With the suggested minor revisions, it will be well-positioned to make a

significant contribution to the field. The transparency of the Delphi process, theoretical justification for the gender constructs, and methodological clarity will further enhance its impact and credibility.

Thank you for your continued dedication to this important work, and I look forward to reviewing the revised submission.
