# Supplementary material for: Initial validity and reliability testing of the SGBA-5
Source: PLoS One. 2025 May 16;20(5):e0323834. doi: 10.1371/journal.pone.0323834 (PMC12084046; doi:10.1371/journal.pone.0323834)
Supplement: Attachment — Submitted filename: Response to Reviewers.docx [file pone.0323834.s006.docx]

We want to thank the Associate Editor for providing us with the opportunity to revise and resubmit this paper. There are several instances where the reviewers have raised concerns that make assumptions about the intent of the proposed SGBA-5 measurement tool and/or the positionality of the authors of this paper that are clearly addressed in the manuscript itself. We have addressed these comments respectfully in our point by point response.

We hope that the Editorial team agrees that this tool and paper can be of significant value to the research community.

Journal Requirements:

1. Please ensure that your manuscript meets PLOS ONE's style requirements, including those for file naming. The PLOS ONE style templates can be found at <https://journals.plos.org/plosone/s/file?id=wjVg/PLOSOne_formatting_sample_main_body.pdf> and <https://journals.plos.org/plosone/s/file?id=ba62/PLOSOne_formatting_sample_title_authors_affiliations.pdf>

Thank you, we have endeavoured to update the manuscript and file names to be in accordance with these guidelines.

2. Please expand the acronym “CIHR” (as indicated in your financial disclosure) so that it states the name of your funders in full. This information should be included in your cover letter; we will change the online submission form on your behalf.

Thank you for doing so. We have updated the cover letter with the financial disclosure statement and expanded the CIHR acronym in that statement.

3. We noted in your submission details that a portion of your manuscript may have been presented or published elsewhere. [An earlier version of this research manuscript was published as a chapter in author AP's master of health science thesis document at Ontario Tech University. A copy of the thesis has been uploaded as a related manuscript file.] Please clarify whether this [conference proceeding or publication] was peer-reviewed and formally published. If this work was previously peer-reviewed and published, in the cover letter please provide the reason that this work does not constitute dual publication and should be included in the current manuscript.

A previous version of this manuscript was included as a chapter in author AP’s master’s thesis and thus is available through Ontario Tech University institutional repository of theses and dissertations. This work has not been formally published or peer-reviewed previously. We have updated the cover letter to reflect his with the following statement:

“An earlier version of this research manuscript was included as a chapter in author AP’s master’s thesis at Ontario Tech University. As part of AP’s thesis defense, the chapter was examined by AP’s thesis committee and the final thesis document is available in Ontario Tech University’s institutional repository eScholar (https://ontariotechu.scholaris.ca/). The manuscript has not been formally peer-reviewed or published and thus does not constitute dual publication.” – Cover Letter

4. Please include captions for your Supporting Information files at the end of your manuscript, and update any in-text citations to match accordingly. Please see our Supporting Information guidelines for more information: <http://journals.plos.org/plosone/s/supporting-information>.

Thank you, we have added the captions to the end of the manuscript and updated the file naming accordingly.

**A note to all reviewers:** It is important to note that the first iteration of any tool cannot be perfect. Throughout our manuscript, we emphasize that further testing is needed and that we are simply presenting *a way*, not *the way* for sex and gender-based analysis in health sciences research.

We appreciate your insights; we hope that our responses clarify the choices we have made. This tool may not be applicable in all types of research, and therefore may not be appropriate for some types of health research. We are open to new tool ideas from the research community and hope that this first version of our tool will be iterated upon.

We hope the reviewers agree that while any one tool will not be the best choice in all situations, the development of novel measurement tools like the SGBA-5 is a move in the right direction.

Reviewer #1

1. Thank you for the opportunity to review this interesting manuscript, which proposes a new tool for measuring sex and 4 aspects of gender in health research studies where sex/gender is not the primary focus. I appreciate this initiative as there is a lack of consensus on how to measure these constructs and a straightforward approach is most likely to be used by researchers in non-SGBA fields. I also appreciate the inclusion of multiple dimensions of gender, as the focus is often on a single aspect (e.g., identity). While I am supportive of this endeavour, I have major concerns with the assumptions and approaches taken in developing this tool, which are described below:

**Response:** Thank you for acknowledging that this is a challenging endeavour; one that many do not attempt because of the charged nature of the topic. We appreciate your comments and thoughts, and hope we have fully addressed them below.

2. The authors have present the gender options on a continuum between "masculine" and "feminine". I disagree with the fundamental assumption that these identities are two ends of a spectrum. Could an individual not score high on both? This format also does not capture that aspects of gender can vary significantly based on context. For example, gendered norms may dictate that a woman adopt a more feminine orientation/roles at home, but more masculine traits/roles in the workplace.

**Response:** We did not assume that the sociocultural concepts of masculinity and femininity are solely two ends of a spectrum. We state that we are using a feminine – masculine relative continuum may be *a way* to measure how sex and gender influences health outcomes across a sample or group.

Please see lines 99-105, and 156-164 of the manuscript as well as pages 8-9 FAQ sections: “Can the values from the SGBA-5’s gendered aspects of health items be generalized?”, and “Aren’t masculinity and femininity separate things?” of the S1 File. [SGBA-5 v1.0 documentation] for further explanation.

3. No testing or validation appears to have been done purposively with individuals who have diverse gender identities. While the authors note that this is not to replace specific investigations with these populations, I would suggest adherence to the principle that designing an effective and accessible tool should begin by designing with/for the most marginalized populations, with the assumption that then it should work for everyone. This appears particularly important given the historic exclusion of gender-diverse individuals from health research, which is often attributed to measurement challenges.

**Response:** One of the main strengths of this tool is that it is INCLUSIVE of gender diverse people. We have added the following lines to help clarify this:

[Methods section:]

“...however, the SGBA-5 is designed so that it can be completed by persons of sex and gender minorities as part of studies where sex or gender are not primary variables.” – Lines 169-171

[Discussion section:]

“A strength of the proposed SGBA-5 tool is that gender-diverse persons’ responses can be analyzed without necessitating the large sample sizes (often an n of 500 or more assuming a 1% proportion) that are required to analyze small-proportion nominal groups.”. – Lines 369-372

Our sample did contain gender diverse individuals. It is also important to note that the main creators of the tool are a non-binary person (AP), and a woman of colour (SD). We agree wholeheartedly with the notion of “nothing about us, without us”. This study reports the initial testing of the SGBA-5, and as such, the authors have emphasized that further testing is needed. The current recommendations to avoid using the SGBA-5 in certain situations reflect that these situations should be evaluated individually in addition to the more general validity and reliability testing processes for the new tool. Those sorts of specific use-case investigations are well outside the scope of initial scale testing. The conservative recommendations to avoid the use of a new tool in certain circumstances is a normal part of scale development and having reasonable limitations put on a measurement tool prior to those specific situations being studied should not be interpreted as that tool never being appropriate for use in those situations. **Not knowing if a measurement tool is appropriate in certain situations is not the same as that tool being inappropriate for use in those situations.**

4. The demographics illustrate that it was a very homogeneous participant group with respect to ethnicity/cultural origin. In fact, in one of the supplemental files, the authors state that the most appropriate use of the SGBA-5 would be in homogenous populations. This appears somewhat problematic to me - could this approach perpetuate the general lack of diversity in health research?

**Response:** All studies are subject to sample selection bias, and most validity/reliability studies do not make the effort to recruit multiple samples. To further clarify our recruitment and sample, we have added the following lines to the manuscript:

[Methods section, clarifying recruitment of older adults]

“...through Ontario Tech University’s Age-Friendly Campus email newsletter and through Facebook advertisements targeting older adults, 55+ years of age in the Durham Region” – Lines 256-258

[Discussion section, addressing sample homogeneity]

“Both the student and older adult samples were relatively homogenous, and thus should not be generalized to all university students or all older adults; However, the reliability results...” – Lines 412-414

Regarding the appropriate uses of the SGBA-5 in the documentation, we reiterate that not knowing if a measurement tool is appropriate in certain situations is not the same as that tool being inappropriate for use in those situations. We hope that the SGBA-5 will support more gender-based analysis in health research. The tool can be used in samples that represent a variety of cultural and ethnic backgrounds, different income levels, and more. Even if a truly random sample selection process were used, some amount homogeneity will occur in many studies samples purely as a result of non-discriminatory limiting factors (i.e., inclusion and exclusion criteria, geographic limitations, smaller sample sizes due to ethical, safety, and practicality concerns, etc.). However, just because any one sample is relatively homogenous does not mean that we as researchers are excused from our responsibility to ensure that the diversity of human health is included in our research.

5. I'm struggling to understand the rationale of ending the Delphi study where the authors did. The gender items did not meet the a priori definition for consensus agreement, and yet they were included anyway. Did anything change over the 3 rounds? Was any feedback given that may have improved the consensus ratings? If the a priori benchmark was to find non-consensus acceptable, what was the rationale of doing the Delphi study? If this was based on inconsistencies in definitions of sex and gender, should definitions have been added to the SGBA-5 measure? I'm also concerned by the hypothesis that this was related to miscommunication on the part of the research team. This paper reads as if it is perhaps being submitted prematurely, and there is more development on the tool is first needed.

**Response:** We ended the Delphi in accordance with pre-determined criteria as stated in Lines 237-240, methodology which is discussed on Lines 194-197. This is also explicitly discussed in the discussion section, Lines 445-448.

To clarify rationale the following lines have been added:

“The purpose of this Delphi Expert Consensus study was to receive feedback on the construct validity of the SGBA-5’s scale items from a sample of Canadian health sciences researchers (this study’s Delphi experts and the most likely initial users of the SGBA-5). This process provided evidence of each SGBA-5 scale items’ adequacy in being able to measure what that item is proposed to measure (an item’s content validity).[16,17] In accordance with threshold of evidence used to initially select the items for inclusion in the SGBA-5, it was decided *a priori* that any major changes (i.e., adding a new item, switching from continuous to ordinal measures, etc.) resulting from the Delphi expert’s feedback must reflect evidence in the current health sciences literature. ” – Lines 198-205

Removing scale items that were neither strongly accepted nor rejected by the Delphi Expert study when those items are grounded in literature would be dubious. This study is an initial assessment of the SGBA-5’s construct validity of health sciences researchers. Even if every item received 100% agreement, that result would only provide evidence for validity, not confirmation that scale items are entirely valid. Scale validity testing is an ongoing process requiring numerous studies (ideally conducted by independent researchers) and requires the measurement tool to be tested in multiple ways and drawing upon the expertise of many different groups. This is a normal part of scale development.

6. I would suggest not using the term "biological sex" if what the researchers are intending to measure is "sex assigned/designated at birth", as gender-affirming medical treatments inherently change an individuals' biology.

**Response:** Thank you for your comment. We are using this term consistently with how the SGBA-5 is designed to aid in assessing differences in health which are influenced by sex (biologically derived) and gender (socio-culturally derived). The limitations of measuring biological sex using “sex assigned at birth” are noted in Lines 131-134, and more broadly in Lines 123-127.

7. Does "North American" mean in the Ethnic/Cultural Origin category include Indigenous populations?

**Response:** The categories listed in the table appear as they did in the survey. Thus, it is possible that Indigenous people identified as North American. We did not have partnerships with Indigenous communities for this research. As per the First Nations principles of ownership, control, access, and possession (OCAP) principles, we cannot make any further claims related to indigenous people with this research.

Reviewer #2:

1. I wanted to thank the authors for contributing to the sex and gender science. The manuscript presents the development and validation of the Sex-and Gender-Based Analysis Tool (SGBA-5) aimed at integrating sex and gender considerations into health research. This tool addresses a gap in the availability of concise, reliable, and valid measurement instruments for assessing sex and gender influences on health. The study utilizes a Delphi consensus method and a test-retest study to evaluate the tool's validity and reliability.

**Response:** Thank you for appreciating the work we’ve undertaken, and for taking the time to review our manuscript. We appreciate your thoughtful comments and have addressed each of them below.

2. Because of the crucial role played by a tool such as this, it is essential to provide as much detail as possible in the development process. Details are needed on the methodology and results for the following steps:

- Initial Design and Development:

(1) extensive reviews of the peer-reviewed and grey literature. Providing details (or reference of a previous publication) on these are necessary, and for example, they will also allow the reviewers and readers to understand why more relevant articles were not referenced in the following statement “The four gender constructs included in the SGBA-5 were chosen because evidence was found in the literature to support their proposed pathway of health impact (gender identity,[17,18] gender expression,[19,20] gender roles,[21,22] and gendered relations[23,24]).

**Response:** We agree with the reviewer that it is critical to clearly articulate the work done to develop the tool. In the section referenced, we comment on the existing literature, and provide our references for each of the constructs. To ensure that the reader is able to delve deeper into our development process, we have now added a reference to the literature review of the master’s thesis of the first author into the introduction section:

“A more detailed analysis of strengths and limitations of existing tools is available elsewhere. [14]” – Lines 75-76

This 50-page review will likely answer most questions readers have regarding the choices and constructs.

3. (2) theoretical foundation for combining a categorical measure of sex and a continuous measure of gender using 4 dimensions; the choice of 100 point-scale

**Response:** The tool does not combine sex and gender, and the analysis appendices provide instructions on how to use the various measures to ensure that researchers should not attempt to come up with a combined score.

The number of gender dimensions included in v1.0 of the SGBA is discussed in Lines 137-142.

The gendered aspect of health items use a 101-point ordinal measure with no assumed true zero value. The rationale is discussed on Lines 157-164, and the results (which further support that rationale) are discussed in Lines 416-420.

4. Delphi Expert Consensus: the criteria for selecting experts, demographic characteristic of participants

**Response:** Our selection criteria were provided in the methods section Lines 211-212 as “researchers who had expertise in conducting health research studies with human participants, and who had been involved in CIHR-funded research in the past”. There were no additional inclusion or exclusion criteria. More context was added:

“The purpose of this Delphi Expert Consensus study was to receive feedback on the construct validity of the SGBA-5’s scale items from a sample of Canadian health sciences researchers (this study’s Delphi experts and the most likely initial users of the SGBA-5). This process provided evidence of each SGBA-5 scale items’ adequacy in being able to measure what that item is proposed to measure (an item’s content validity).[16,17] In accordance with threshold of evidence used to initially select the items for inclusion in the SGBA-5, it was decided *a priori* that any major changes (i.e., adding a new item, switching from continuous to ordinal measures, etc.) resulting from the Delphi expert’s feedback must reflect evidence in the current health sciences literature.” – Lines 198-205

With regards to the demographics, we did not obtain this information as the population of health sciences researchers in Canada is too small to report meaningful descriptive statistics while maintaining participant anonymity.

5. Test-Retest Study: Recruitment of participants for the older adult arm

**Response:** Thank you for bringing this to our attention. We have added the following details regarding recruitment to the manuscript:

“...through Ontario Tech University’s Age-Friendly Campus email newsletter and through Facebook advertisements targeting older adults, 55+ years of age in the Durham Region” – Lines 256-258

6. There are two main study designs in this manuscript. At least for the Delphi method, I would recommend that the authors use a reporting guideline for better structure and completeness (e.g. CREDES, Spranger J et al. 2022)

**Response:** Additional information on the rationale of the Delphi study in the context of initial scale development was added:

“The purpose of this Delphi Expert Consensus study was to receive feedback on the construct validity of the SGBA-5’s scale items from a sample of Canadian health sciences researchers (this study’s Delphi experts and the most likely initial users of the SGBA-5). This process provided evidence of each SGBA-5 scale items’ adequacy in being able to measure what that item is proposed to measure (an item’s content validity).[16,17] In accordance with threshold of evidence used to initially select the items for inclusion in the SGBA-5, it was decided *a priori* that any major changes (i.e., adding a new item, switching from continuous to ordinal measures, etc.) resulting from the Delphi expert’s feedback must reflect evidence in the current health sciences literature.” – Lines 198-205

While we appreciate the references provided, they are for use in creating clinical consensus, not for initial content validity assessment of a novel measurement tool.

7. Introduction: -Page 5, lines 70-73, references to existing tools are needed here

**Response:** Lines 70-73 provide a summary of critiques that have been applied to existing scales and those critiques have been cited. We have also added the following resource for those who wish to delve deeper on this topic:

“A more detailed analysis of strengths and limitations of existing tools is available elsewhere. [14]” – Lines 75-76

(This references author AP’s thesis which contains more detailed exploration of existing measures in health sciences).

8. Page 5, lines 74-79, Please also mentioned that a two-step measure of gender has been the recommended approach when using nominal categorizing response options (National Academies of Sciences, Engineering, and Medicine. Measuring Sex, Gender Identity, and Sexual Orientation. Washington, DC: The National Academies Press; 2022.)

**Response:** Thank you. The following has been added: [pre-existing text in square brackets]

“For example, in 2022 the National Academy of Sciences (US) recommended that researchers use a two-step method of nominal categorical responses where participants report their sex-assigned at birth and gender identity separately.[15] The recommendation of the two-step method has evidence supporting its validity and reliability for use in population-level and census questionnaires.[15–17] Unfortunately, [these kinds of categorical indicator variables] can only provide meaningful differentiation between sex-based and gender-based effects in studies that have very large sample sizes, an inherent limitations of using [disaggregation analyses.] [Disaggregation]-based [analyses do not allow for the level of detailed insight that is possible from more complex conceptualizations of sex and gender – which require more intricate scale measures or qualitative work.[18] Thus,] the use of nominal categories and [disaggregated analysis] in SGBA [can provide] some [insight into differences between sex and/or gender categorizations] in large, population-scale surveys however, they cannot provide the level of granular detail needed for investigation of [the multidimensional aspects and within-category variation that is inherent in social constructs like gender.[6]] – Lines	79-91

9. Page 5, lines 79-83: the expression sex/gender has different meanings, please provide the rationale for using it here.

**Response:** Thank you for pointing this out. To ensure clarity, we have edited “sex/gender” to “sex and/or gender” throughout the manuscript.

10. Page 6, lines 100-103: I am expecting to see some references to existing tools drawn from their extensive literature review.

**Response:** We have added a non-exhaustive list of references to existing tools and/or reviews of those tools to this section. Furthermore, we have added a reference to the thesis that provides a more robust and comprehensive literature review.

11. Discussion: Mention of the limitation regarding the method of expert selection is right; the authors would discuss the fact that the tool excludes minoritized and gender diverse people for there doesn't seem to be an option for them.

**Response:** One of the main strengths of this tool is that it is INCLUSIVE of gender diverse people. We have added the following lines to help clarify this:

[Methods section:]

“...however, the SGBA-5 is designed so that it can be completed by persons of sex and gender minorities as part of studies where sex or gender are not primary variables.” – Lines 169-171

[Discussion section:]

“A strength of the proposed SGBA-5 tool is that gender-diverse persons’ responses can be analyzed without necessitating the large sample sizes (often an n of 500 or more assuming a 1% proportion) that are required to analyze small-proportion nominal groups.”. – Lines 369-372

12. There are two main study designs in this manuscript. At least for the Delphi method, I would recommend that the authors use a reporting guideline for better structure and completeness (e.g. CREDES, Spranger J et al. 2022). The manuscript would benefit from a more structured layout E.g. Page 8, pages 134-139: I am trying to figure out what might be the best structure for this paper? I think this text should come earlier or later in the discussion.

**Response:** Thank you for your support. We agree that there is a lot of information to present in one paper. We have tried to follow guidelines and improve clarity of process throughout the manuscript. We have created a figure to begin the methods section that we hope will help with this as well (Lines 109 – 110).

Fig1. Visual representation of the cyclical process of novel scale development. (pasted below with caption following)

“This diagram shows the steps involved in novel scale creation, beginning with Item Development and then progressing to an ongoing cycle of Scale Development and Scale Assessment. This cycle of Scale Development and Scale Assessment continues alongside use of the scale to ensure the scale’s validity and reliability as well as assess the suitability of the scale’s use in different contexts. The diagram also indicates which step in novel scale development each component of the initial design and testing of the SGBA-5 described in this paper are connected to.”

13. There are minor grammatical errors and punctuation issues, e.g. "SGBA-5 tool demonstrated reliability for all items and validity of the biological sex item" could be more clearly written.

**Response:** Thank you for pointing this out. We have edited the manuscript thoroughly and hope we have corrected all typographical issues.

Reviewer #3:

1. the article deals with an important topic: how sex/gender can be recorded in health studies that do not primarily focus on sex/gender. To understand this more deeply, it would be helpful to explain in which aspects of health the respective dimensions surveyed can make a contribution. In my view, simply referring to studies is not sufficient to justify this.

**Response:** Thank you for acknowledging the importance of this work. With regards to the issue of “which aspects of health” this is a difficult one to respond to as the evidence suggests that gender influences a multitude of health issues, but the lack of tools available has made it challenging to appropriately study.

To ensure that the reader is able to delve deeper into our development process, we have added a reference to the literature review of the master’s thesis of the first author into the introduction section:

“A more detailed analysis of strengths and limitations of existing tools is available elsewhere. [14]” – Lines 75-76

This 50-page review will likely answer most questions readers have regarding the choices and constructs.

2. There is also a lack of more precise information on who took part in the delphi study. Depending on who takes part in such a study will also influence the content of the feedback. Does the feedback from people who deal specifically with gender issues differ from those who do not? In my view, it would make sense to involve the expertise of gender researchers in the content of the questions and to ask health scientists about the practicability of the survey instruments.

**Response:** Thank you for this suggestion. Scale validity testing requires many studies with many different groups such as the suggested gender researchers. There is no one group that could possibly determine a measurement tools validity alone, thus the selection of health researchers (or any other group) does not preclude the expertise of other groups. This is a normal part of scale development.

To clarify rationale the following lines have been added:

“The purpose of this Delphi Expert Consensus study was to receive feedback on the construct validity of the SGBA-5’s scale items from a sample of Canadian health sciences researchers (this study’s Delphi experts and the most likely initial users of the SGBA-5). This process provided evidence of each SGBA-5 scale items’ adequacy in being able to measure what that item is proposed to measure (an item’s content validity).[16,17] In accordance with threshold of evidence used to initially select the items for inclusion in the SGBA-5, it was decided *a priori* that any major changes (i.e., adding a new item, switching from continuous to ordinal measures, etc.) resulting from the Delphi expert’s feedback must reflect evidence in the current health sciences literature. ” –Lines 198-205

3. It is precisely in the interpretation of the items that theoretical gaps appear, which again refer to my first point: Why was which gender dimension surveyed? How do these influence health? How can the milieu-specific understanding of gender be captured?

**Response:** Thank you for this comment. The reasoning for the selection of gender dimensions included in v1.0 of the SGBA is discussed in Lines 116-124. For more detail on how these dimensions are operationalized please see Lines 99-105, and 156-164 of the manuscript as well as pages 8-9 FAQ sections: “Can the values from the SGBA-5’s gendered aspects of health items be generalized?”, and “Aren’t masculinity and femininity separate things?” of the S1 File. [SGBA-5 v1.0 documentation].

To ensure that the reader can delve deeper into our development process, we have added a reference to the literature review of the master’s thesis of the first author into the introduction section:

“A more detailed analysis of strengths and limitations of existing tools is available elsewhere. [14]” – Lines 75-76

This 50-page review will likely answer most questions readers have regarding the choices and constructs.

4. What exactly does female-male mean? These binary poles are linked to social stereotypes and ultimately reproduce them.

**Response:** We are unable to find any occurrence of “female-male” (with or without spaces) in our manuscript. If the reviewer is simply referring to the terms of male and female, those two terms are used in the context of biological sex, and do not appear in our manuscript without also mentioning intersex (e.g. Line 128).

5. In order to understand how the survey instrument can be used, it is helpful not only to refer to another publication that has worked with this survey instrument. At least a brief summary would be helpful.

**Response:** We are not clear on what the reviewer is asking for here. This is a new instrument and has not been previously used.

6. In my view, it cannot be concluded from the Delphi results (consensus is: no consensus!) that the gender items can be used without reservation.

**Response:** We agree with the reviewer, we did not and would not make such a claim. We are unaware of any measurement tool or scale item that should be used without reservation based solely upon initial validity and reliability testing.
