# Supplementary material for: Initial validity and reliability testing of the SGBA-5
Source: PLoS One. 2025 May 16;20(5):e0323834. doi: 10.1371/journal.pone.0323834 (PMC12084046; doi:10.1371/journal.pone.0323834)
Supplement: Attachment — Submitted filename: Reviwer report 29_09_2024.docx [file pone.0323834.s005.docx]

**Review Report for PLOS One Manuscript: Initial Validity and Reliability Testing of the SGBA-5**

**Manuscript Number:** PONE-D-24-12283R1
**Title:** Initial Validity and Reliability Testing of the SGBA-5
**Reviewed by:** Single blinded review

**General Comments:**

The manuscript under review presents the development and initial validation of the SGBA-5, a tool intended to facilitate sex- and gender-based analysis (SGBA) in health sciences research. This is a valuable initiative, addressing the need for concise and reliable tools in this area, particularly for studies where sex or gender are not primary variables of interest.

The authors have provided responses to prior reviews and made significant revisions. The manuscript is generally well-structured, and the authors have done a good job of outlining the importance of their work. However, several areas still require further clarification or revision, particularly regarding the consensus-building process in the Delphi study and the tool’s applicability across diverse populations.

**Major Issues:**

1. *Delphi Methodology and Consensus***:**

The authors have set a consensus threshold of 75% for the Delphi study, but the gendered items (e.g., identity, roles, expression) did not meet this threshold. The decision to include these items despite the lack of consensus is not fully justified. While the authors acknowledge that scale development is an ongoing process, additional clarity on why the Delphi process was halted after three rounds is necessary. Was there significant feedback across rounds? Were the disagreements rooted in theoretical differences or practical concerns?

**Recommendation:** The inclusion of gendered items requires stronger justification, and a deeper analysis of the feedback provided by the experts would be beneficial. A clearer explanation of why consensus was not achieved and how the authors plan to address this in future iterations would strengthen the paper.

2. *Diversity of the Sample:*

The manuscript notes that the study sample, particularly in the Delphi study, was relatively homogeneous in terms of ethnicity and cultural background. While this limitation is acknowledged, the lack of diversity in the sample may impact the tool’s broader applicability, particularly with respect to gender-diverse populations. The authors emphasize that future testing is needed in more diverse groups, but this point could be highlighted more strongly.

**Recommendation:** The limitations of the sample, especially in terms of gender diversity, should be more explicitly discussed. Future versions of the SGBA-5 should prioritize testing with gender-diverse individuals to ensure inclusivity and generalizability.

3. *Theoretical Justification for Gender Constructs:*

The manuscript introduces four gender constructs (identity, expression, roles, relations) but provides limited theoretical explanation for their selection. Although relevant references are included, a more thorough discussion of how these constructs influence health outcomes is needed. This would provide a stronger foundation for the tool’s use in health research.

**Recommendation:** A more detailed exploration of the theoretical basis for the gender constructs is needed. Specifically, the authors should expand on how these constructs have been operationalized in previous research and how they are expected to interact with health outcomes.

4. *Terminology:*

The manuscript uses the term "biological sex" throughout, although it is noted that gender-affirming treatments can alter biological characteristics. Several reviewers recommended the use of "sex assigned at birth" as a more accurate term in this context. While the authors have addressed this in part, a more inclusive approach to terminology would enhance the clarity and appropriateness of the tool.

**Recommendation:** Consider revising the terminology to "sex assigned at birth" to better account for gender-affirming treatments and evolving biological characteristics.

5. *Reliability and Validity Testing***:**

The test-retest reliability results are promising, with strong coefficients for both the student and older adult arms. However, the inclusion of gendered items without full consensus from the Delphi study raises concerns about the validity of these items. Further evidence is needed to justify their inclusion.

**Recommendation:** Future studies should focus on re-evaluating these gender constructs, particularly in more diverse populations. Additionally, a stronger theoretical justification for the constructs would enhance confidence in their validity.

**Minor Issues:**

1. *Grammatical and Stylistic Improvements:*

While the manuscript is generally well-written, there are areas where clarity can be improved, particularly in the explanation of key concepts such as sex and gender. Several typographical errors and unclear phrasings should be addressed in a thorough revision.

**Recommendation:** A final proofreading and revision of key sections for clarity and consistency would improve the overall quality of the manuscript.

2. *Methodology Clarification***:**

The inclusion of Figure 1, which illustrates the cyclical process of scale development, is helpful in clarifying the iterative nature of the tool's development. However, further detail on how feedback was integrated into the process and how decisions were made at each stage would be useful.

**Recommendation:** Additional detail on the methodology, particularly regarding feedback integration, would enhance the clarity of the manuscript.

3. *Responses to Reviewers***:**

The authors have provided detailed responses to many reviewer concerns, but some issues, particularly around the Delphi consensus and sample diversity, require further elaboration. Addressing these points more thoroughly in future revisions would strengthen the manuscript.

**Conclusion:**

The development of the SGBA-5 is a valuable contribution to the field, offering a novel tool for integrating sex and gender considerations into health research. However, there are several areas that require further revision, particularly regarding the theoretical foundation of the tool, the diversity of the sample, and the consensus-building process in the Delphi study. With further refinement, this tool has the potential to make a significant impact on health research.

**Recommendation:**

Major Revisions
