# Supplementary material for: Initial validity and reliability testing of the SGBA-5
Source: PLoS One. 2025 May 16;20(5):e0323834. doi: 10.1371/journal.pone.0323834 (PMC12084046; doi:10.1371/journal.pone.0323834)
Supplement: Attachment — Submitted filename: Response_to_Reviewers_auresp_2.docx [file pone.0323834.s008.docx]

## EDITOR

Thank you for giving us the opportunity to further revise this paper. We have addressed each of the reviewer comments below, and hope that you agree that we have gone above and beyond in defending and describing this work simply because of the topic of the work. As we mentioned to the Editor and Reviewers in the previous round, this paper presents an initial assessment of a novel scale which includes two assessments of validity; establishment of face validity from the literature and small group feedback as well as the Delphi study which further investigates one aspect of the SGBA-5’s content validity in a sample of Canadian health researchers. This work also includes test-retest reliability studies in two distinct population groups. The scope of this paper is already broad, and we acknowledge this tool can benefit from future validity and reliability testing with more participants.

There is a significant gap in the tools available to researchers that is holding back our understanding of the influence of sex and gender on health. This tool is a first iteration that we hope will spark further advancement in the field.

## Reviewer #1:

1. Thank you for the opportunity to re-review this manuscript. I appreciate that further validation is needed beyond any initial tool. I would like to clarify the intention behind some of my original comments: My concern is that once a tool is available in the public domain, particularly a tool that is unlikely to be the primary measure of a study, that many researchers would use it “as is” rather than conducting their own reliability/validity research for their specific sample. This is why I suggested more purposive recruitment efforts towards marginalized/underrepresented populations in health research if the authors are indeed hoping for it to be broadly applicable across the Canadian health research landscape. I would hope that a more diverse validation/reliability study would help increase the appropriate use of the tool AND also exemplify a more inclusive way of conducting these types of studies.

**Response:** Thank you, we agree with your concern. It is not practical to expect that each researcher conducts validity and reliability testing on each measurement tool they use. The testing of multiple aspects of validity and reliability properly across diverse populations is important and is a considerably larger task than the scope of a single paper. We have added the following limitation to the discussion section to address this concern:

“Further evaluation of the SGBA-5 should investigate the different aspects of scale validity and reliability across diverse population groups.” (Lines 433-445)

2. The authors’ response states that “We did not assume that the sociocultural concepts of masculinity and femininity are solely two ends of a spectrum.” If that is the case, I am unclear on why it would be presented as such in the questionnaire. It is also not clear how is a score at the middle of the spectrum interpreted – does this mean the individual is high on femininity and masculinity, or low on both?

**Response:** We have added a sentence to address the choice of using of the continuum in lines 150-153 of the manuscript. The sentence added reads as follows:
{previously existing text in curled brackets}.

As the CIHR Institute of Gender and Health notes in its definitions of sex and gender in the context of health research, “[gender] is not confined to a binary (girl/woman, boy/man) nor is it static; it exists along a continuum and can change over time”.[1] To attempt to capture more of this variation than nominal items alone could provide, {feminine – masculine analogue (continuous) scales were used…} (Lines 150-153)

The issue of interpretation is covered in the supplementary example analysis provided and in the FAQ section of supplementary File 1. We have provided a quick summary here as well.

Since the SGBA-5 is designed to assess group-level differences, the “score” of the continuous gender measures is interpreted as an ordinal series with the middle not assumed to be a true “0” point. In practice that means that health researchers would use statistical tests of difference like a t-test to see if participant’s gendered aspects of health item ‘scores’ differ between study groups (e.g., intervention group vs. control group, or participants with an outcome vs. participants without that outcome). This provides researchers insight into whether these gendered aspects of health could be confounding the study’s results (and ideally then spur further research specifically into how sex or gender may be affecting that area of health).

3. In their response letter the authors indicated that gender-diverse individuals were included in their sample, but this data does not appear to be reported anywhere in the manuscript (or do the authors mean that gender-diverse individuals were not excluded from participation but that gender identity data was not collected?). Please clarify.

**Response:** The SGBA-5 itself reports information on gender diversity across each of the 4 gendered aspects of health included. We did not also collect nominal gender identity due the exclusionary nature of disaggregation analysis in sample sizes we had, which are addressed in Lines 79-92 of the manuscript.

Reviewer #4:

*General Comments:*

1. The manuscript under review presents the development and initial validation of the SGBA-5, a tool intended to facilitate sex- and gender-based analysis (SGBA) in health sciences research. This is a valuable initiative, addressing the need for concise and reliable tools in this area, particularly for studies where sex or gender are not primary variables of interest.
   The authors have provided responses to prior reviews and made significant revisions. The manuscript is generally well-structured, and the authors have done a good job of outlining the importance of their work. However, several areas still require further clarification or revision, particularly regarding the consensus-building process in the Delphi study and the tool’s applicability across diverse populations.

**Response:** Thank you for your appreciation and for the time you’ve taken to review our work.

*Major Issues:*

2. Delphi Methodology and Consensus:
   The authors have set a consensus threshold of 75% for the Delphi study, but the gendered items (e.g., identity, roles, expression) did not meet this threshold. The decision to include these items despite the lack of consensus is not fully justified. While the authors acknowledge that scale development is an ongoing process, additional clarity on why the Delphi process was halted after three rounds is necessary. Was there significant feedback across rounds? Were the disagreements rooted in theoretical differences or practical concerns?
   *Recommendation:* The inclusion of gendered items requires stronger justification, and a deeper analysis of the feedback provided by the experts would be beneficial. A clearer explanation of why consensus was not achieved and how the authors plan to address this in future iterations would strengthen the paper.

**Response:** Thank you for giving us the opportunity to further clarify this issue. The Delphi consensus threshold was set at a conservative 75%. We did this deliberately as we are creating a novel scale. The fact that our sample of experts did not arrive at the 75% approval threshold for an item does not solely reflect on the items’ construction. It is also a reflection that there is lack of consensus about how to measure how gender impacts health among experts. As we state in Lines 100-101, [the SGBA-5 aims to be] *one* *way* to conduct SGBA in health research studies based on current evidence of how sex and gender influence health but is certainly not *the* *only* *way* to do so. In fact, the enough of the optional comments we received during the first round were offensive and ignorant that we felt compelled as a research team to specify our positionality in the second round and clarify that we had good intentions and were researching in good faith. Understandably, we did not include those comments in this paper.

In terms of why the Delphi was halted, this is addressed in Lines 173-174 where we state that the Delphi study would be halted once researchers reach any stable consensus (including but not limited to consensus approval). Lines 213-215 then specify the Delphi’s study non-approval consensus threshold (difference in between-round CV of < .15). This threshold is also included in the foot notes of Table 2 (lines 296-297).

3. Diversity of the Sample:
   The manuscript notes that the study sample, particularly in the Delphi study, was relatively homogeneous in terms of ethnicity and cultural background. While this limitation is acknowledged, the lack of diversity in the sample may impact the tool’s broader applicability, particularly with respect to gender-diverse populations. The authors emphasize that future testing is needed in more diverse groups, but this point could be highlighted more strongly.
   *Recommendation:* The limitations of the sample, especially in terms of gender diversity, should be more explicitly discussed. Future versions of the SGBA-5 should prioritize testing with gender-diverse individuals to ensure inclusivity and generalizability.

**Response:**  Thank you, we agree. We have added the following limitation to the discussion section:

“Further evaluation of the SGBA-5 should investigate the different aspects of scale validity and reliability across diverse population groups.” (Lines 433-445)

4. Theoretical Justification for Gender Constructs:
   The manuscript introduces four gender constructs (identity, expression, roles, relations) but provides limited theoretical explanation for their selection. Although relevant references are included, a more thorough discussion of how these constructs influence health outcomes is needed. This would provide a stronger foundation for the tool’s use in health research.
   *Recommendation:* A more detailed exploration of the theoretical basis for the gender constructs is needed. Specifically, the authors should expand on how these constructs have been operationalized in previous research and how they are expected to interact with health outcomes.

**Response:** We agree with the reviewer that a detailed exploration of the theory and its potential implications is important. Indeed, the lead author did this as part of their thesis. However, reporting that literature review is outside the scope of a paper reporting on the initial validity and reliability testing of a novel scale. Further justification of the items can be found in AP’s thesis, which is cited in line 83 of the manuscript, full reference on Lines 493-494).

5. Terminology:
   The manuscript uses the term "biological sex" throughout, although it is noted that gender-affirming treatments can alter biological characteristics. Several reviewers recommended the use of "sex assigned at birth" as a more accurate term in this context. While the authors have addressed this in part, a more inclusive approach to terminology would enhance the clarity and appropriateness of the tool.
   *Recommendation:* Consider revising the terminology to "sex assigned at birth" to better account for gender-affirming treatments and evolving biological characteristics.

**Response:** Thank you for your comment. We are using this term consistently with how the SGBA-5 is designed to aid in assessing differences in health which are influenced by sex (biologically derived) and gender (socio-culturally derived). The limitations of discussing the range of physical and physiological mechanisms through which sex can impact health solely through the lens of “sex assigned at birth” are noted in Lines 131-134, and more broadly in Lines 123-127. Furthermore, as Canadian researchers, we are aligning our work with national funding agency policies.

6. Reliability and Validity Testing:
   The test-retest reliability results are promising, with strong coefficients for both the student and older adult arms. However, the inclusion of gendered items without full consensus from the Delphi study raises concerns about the validity of these items. Further evidence is needed to justify their inclusion.
   *Recommendation:* Future studies should focus on re-evaluating these gender constructs, particularly in more diverse populations. Additionally, a stronger theoretical justification for the constructs would enhance confidence in their validity.

**Response:** This study represents initial investigation of a novel measurement tool, with the Delphi study specifically representing initial evaluation of content validity from the perspectives of a group of health researchers (paraphrased from Lines 194-196). Given the lack of previously validated items from which to compare the content, construct, or face validity of the novel SGBA-5 it would be inappropriate to overgeneralize the results based on a single study alone. We have noted the opportunities for future research, and agree with the reviewer that our initial reliability data are promising. Finally, we have referenced AP’s thesis for the readers who choose to learn more about this scale and topic.

*Minor Issues:*

7. Grammatical and Stylistic Improvements:
   While the manuscript is generally well-written, there are areas where clarity can be improved, particularly in the explanation of key concepts such as sex and gender. Several typographical errors and unclear phrasings should be addressed in a thorough revision.
   *Recommendation:* A final proofreading and revision of key sections for clarity and consistency would improve the overall quality of the manuscript.

**Response:** We have carefully reviewed the manuscript and hope we have identified and addressed all issues.

8. Methodology Clarification:
   The inclusion of Figure 1, which illustrates the cyclical process of scale development, is helpful in clarifying the iterative nature of the tool's development. However, further detail on how feedback was integrated into the process and how decisions were made at each stage would be useful.
   *Recommendation:* Additional detail on the methodology, particularly regarding feedback integration, would enhance the clarity of the manuscript.

**Response:** Thank you. We have clarified the optional feedback we received from the Delphi experts in adding the following lines to the manuscript:

“The optional feedback provided by the Delphi experts did not provide any new insights into or constructive critique of the SGBA-5.” (Lines 314-315)

[Please note: we also address this issue in our response to comment 2.]

9. Responses to Reviewers:
   The authors have provided detailed responses to many reviewer concerns, but some issues, particularly around the Delphi consensus and sample diversity, require further elaboration. Addressing these points more thoroughly in future revisions would strengthen the manuscript.

**Response:** Thank you for your feedback, and comments above. We hope we have addressed them to your satisfaction, and that you agree the manuscript is clearer as a result.

*Conclusion:*

10. The development of the SGBA-5 is a valuable contribution to the field, offering a novel tool for integrating sex and gender considerations into health research. However, there are several areas that require further revision, particularly regarding the theoretical foundation of the tool, the diversity of the sample, and the consensus-building process in the Delphi study. With further refinement, this tool has the potential to make a significant impact on health research.

**Response:** Thank you again for taking the time to review this manuscript, and for acknowledging the importance of our work. We hope we have been able to address your concerns in our responses.

Reviewer #5:

1. I Thank The authors for this interesting and timely paper on a tool for sex and gender-based analysis.

**Response:** Thank you for your support and for the time you’ve taken to review our work.

2. I have my concerns of using the feminine- masculine continuum, especially in expressing the gender-expression, gender-roles and gender-relations, based on my experience with the community. Since, the gender-roles and gender-relations and even expression may differ based on the context and would vary.
   However, the test-re-test assessment shows that at least a part of the population (older adults and university students) identify it correctly. I wonder whether it would be the same for the working population as well.

Nevertheless, as the authors have mentioned, this is only the first assessment and further studies are required in a more generalized sample to assess its validity.
I suggest the authors to emphasize the fact for the readers that this tool needs further studies in more generalized sample(s) prior to its use for SGBA.

**Response:** With regards to the continuum, we have added the following sentence to lines 150-153 of the manuscript:
{previously existing text in curled brackets}.

As the CIHR Institute of Gender and Health notes in its definitions of sex and gender in the context of health research, “[gender] is not confined to a binary (girl/woman, boy/man) nor is it static; it exists along a continuum and can change over time”.[1] To attempt to capture more of this variation than nominal items alone could provide, {feminine – masculine analogue (continuous) scales were used…} (Lines 150-153)

Thank you for acknowledging that this is a first iteration of the tool. We definitely agree that further testing is required. We have added the following limitation to the discussion section:

“Further evaluation of the SGBA-5 should investigate the different aspects of scale validity and reliability across diverse population groups.” (Lines 433-445)

3. The corrections that the authors have made based on the other reviewers' comments/suggestions are acceptable to me.

**Response:** Thank you, we hope we have been able to address any question or concerns you have.
