# Supplementary material for: Initial validity and reliability testing of the SGBA-5
Source: PLoS One. 2025 May 16;20(5):e0323834. doi: 10.1371/journal.pone.0323834 (PMC12084046; doi:10.1371/journal.pone.0323834)
Supplement: Attachment — Submitted filename: Response_to_Reviewers_auresp_3.docx [file pone.0323834.s009.docx]

## EDITOR

Thank you for the opportunity to respond to the reviewer’s comments. As you will note from our responses, the reviewer is no longer identifying new issues, rather, they are going back to issues that we have already addressed in the previous two rounds of revisions. Furthermore, they appear to have limited understanding of the methods we have employed, justified, and thoroughly explained in multiple places in the manuscript. Finally, we do not feel we can ever satisfy a reviewer’s personal preference for language and terminology. We have used terms that we believe are appropriate and are grounded in research.

We hope that you agree that the paper is ready for publication in its current form as a result of the rigorous review process it has undergone. This has been a long review process (original submission March 2024), so we look forward to a final decision on this submission soon.

REVIEWER # 4

1. Thank you for your thoughtful revisions and responses to the previous round of comments. The manuscript reflects substantial effort in addressing key concerns, particularly in acknowledging the limitations of the sample diversity, providing insights into the reliability testing, and justifying the inclusion of the gendered constructs. These updates strengthen the study's foundational contribution as a preliminary step in the development and validation of the SGBA-5 tool. However, some areas still require further clarification and refinement to ensure the manuscript meets its full potential. Below are the comments and suggestions for further improvements.

**Response:** Thank you for taking the time to review our work again, and for your kind words regarding our novel work.

### Delphi Study and Consensus

2. The decision to halt the Delphi process after three rounds and include gendered items despite not meeting the 75% consensus threshold is partially justified. However, the rationale could be more robust. Specifically:
   a. Provide a more detailed analysis of the expert disagreements and clarify whether the non-consensus stemmed from theoretical differences or practical concerns.

**Response:** Unfortunately, it appears there is some misunderstanding. This study asked the experts to rate the SGBA-5 items validity for use in health research on 1 to 5 Likert scale (the primary focus of this study), then experts had the option to provide qualitative comments regarding the items, some of whom did. As stated in the manuscript, the purpose of this was to provide “feedback on the construct validity”, not to provide insight on philosophical or theoretical differences of health sciences researchers.

This is certainly an area with potential for further research (as is noted in the discussion section in lines 366-375) but is outside the scope of the initial validity and reliability testing reported in this paper.

As we have acknowledged, in the manuscript “The resulting tool is proposed as **one way** to conduct SGBA in health research studies based on current evidence of how sex and gender influence health but is certainly not the only way to do so.”. Surely, there are a multitude of ways in which a new tool can be designed; the SGBA-5 is a result of the literature, methods, and approaches we chose to implement, all of which are evidence-based and justified throughout the manuscript.

b. If feasible, summarize the key optional feedback received (even if it was not constructive) to illustrate the range of expert perspectives.

**Response:** A qualitative analysis of the health researcher’s optional feedback is outside of the scope of this study. As we mentioned in the 2^nd^ round of reviewer responses, the lines 314-315 were updated in the manuscript to clarify that no additional insight into the SGBA-5’s item’s validity was provided in the qualitative comments.

“The optional feedback provided by the Delphi experts did not provide any new insights into or constructive critique of the SGBA-5.” (Lines 314-315)

### Sample Diversity

3. While the manuscript acknowledges the homogeneity of the Delphi panel and test-retest populations, the implications of this limitation should be emphasized more strongly.
   a. For example, discuss how the findings might differ in gender-diverse or underrepresented populations and suggest specific future research directions to address this gap.

**Response:** This limitation has been addressed in response to a previous reviewer. Specifically, details can be found in the discussion section in lines 414-424.

It would be highly inappropriate of us to speculate on how the findings might differ in a gender-diverse or under-representative sample. To be clear, this tool has been explicitly designed as a secondary measure for health research in general populations (see initial design and development section of the manuscript, lines 151-157) – to conduct the *initial* validity and reliability tests *in situations where the tool is not designed to work* would be inconsistent with expectations of tool development research.

We agree that studying the SGBA-5 with these groups specifically should be done in the future. Further testing is also needed using larger, more diverse samples to study its potential generalizability (this version of the tool is not assumed to be generalizable) but such research is not a reasonable starting pointing for assessing a novel measurement item.

### Theoretical Framework

4. The four gender constructs (identity, expression, roles, relations) are an important aspect of the SGBA-5, yet their theoretical justification could be expanded.
   a. Include a brief discussion in the main text explaining why these constructs were chosen and how they influence health outcomes. While the reference to the thesis is helpful, a concise summary would provide more immediate context for readers.

**Response:** Sources in addition to the thesis work are cited throughout the initial design and development section (e.g., lines 100-113, and 128-129). Including more detailed description of the constructs is beyond what is typically included in a manuscript on testing validity and reliability. If the Editor wishes, we can add an appendix to the manuscript to add further detail.

### Terminology

5. The consistent use of "biological sex" aligns with Canadian research standards; however, complementing this with "sex assigned at birth" where appropriate would enhance inclusivity and align with best practices in gender-affirming contexts.

**Response:** We appreciate your input on this, but have chosen to be consistent with use of our terminology to avoid confusion to the reader.

### Clarity and Style

6. While the manuscript is generally well-written, certain sections (e.g., discussion and methodology) would benefit from additional clarity. Simplify complex sentences and ensure consistent terminology throughout the text.
   a. A final proofreading pass is recommended to ensure grammatical accuracy and improve readability.

**Response:** Thank you, we have reviewed the manuscript.

### Methodology Transparency

7. The integration of feedback from the Delphi study could be described in greater detail.
   a. For example, specify how disagreements or diverse opinions were handled in the revision process, even if optional feedback provided little constructive insight.

**Response:** This has been answered in our response to question #2.

### Supplementary Materials

8. Key details from supplementary materials, such as the example analysis and methodological rationale, should be summarized briefly in the main text to make the paper more self-contained.

**Response:** Including such details is not a typical in a manuscript on testing validity and reliability, however if the editor requests wishes it can be added to the manuscript as well. Alternatively, we can remove this supplementary material if it is distracting from the main purpose of the manuscript i.e. the initial validity and reliability.

### Conclusion

9. This manuscript represents an important step in advancing tools for SGBA in health research. With the suggested minor revisions, it will be well-positioned to make a significant contribution to the field. The transparency of the Delphi process, theoretical justification for the gender constructs, and methodological clarity will further enhance its impact and credibility.
   Thank you for your continued dedication to this important work, and I look forward to reviewing the revised submission.

**Response:** Thank you again for taking the time to review this manuscript, and for acknowledging the importance of our work. We hope we have been able to address your concerns in our responses.
