# Supplementary material for: Initial validity and reliability testing of the SGBA-5
Source: PLoS One. 2025 May 16;20(5):e0323834. doi: 10.1371/journal.pone.0323834 (PMC12084046; doi:10.1371/journal.pone.0323834)
Supplement: Peer review history

# Author comment: Author response to Decision Letter 0

**DOI:** 10.1371/journal.pone.0323834.r001

1 Apr 2024

# Peer review: Decision Letter 0

**DOI:** 10.1371/journal.pone.0323834.r002

**Authors:** Jennifer Tucker

1 Jul 2024

PONE-D-24-12283Initial Validity and Reliability Testing of the SGBA-5PLOS ONE

Dear Dr. Putman,

Thank you for submitting your manuscript to PLOS ONE. After careful consideration, we feel that it has merit but does not fully meet PLOS ONE’s publication criteria as it currently stands. Therefore, we invite you to submit a revised version of the manuscript that addresses the points raised during the review process.

Please carefully address the revision requests from all three reviewers, including Reviewer 1 who raises serious concerns regarding the definitions and binary nature of the sliding scale within the suggested SGBA-5, and queries regarding the conclusions from the Delphi study. 

Please submit your revised manuscript by Aug 12 2024 11:59PM. If you will need more time than this to complete your revisions, please reply to this message or contact the journal office at plosone@plos.org . When you're ready to submit your revision, log on to https://www.editorialmanager.com/pone/ and select the 'Submissions Needing Revision' folder to locate your manuscript file.

Please include the following items when submitting your revised manuscript:

- A rebuttal letter that responds to each point raised by the academic editor and reviewer(s). You should upload this letter as a separate file labeled 'Response to Reviewers'.
- A marked-up copy of your manuscript that highlights changes made to the original version. You should upload this as a separate file labeled 'Revised Manuscript with Track Changes'.
- An unmarked version of your revised paper without tracked changes. You should upload this as a separate file labeled 'Manuscript'.

If you would like to make changes to your financial disclosure, please include your updated statement in your cover letter. Guidelines for resubmitting your figure files are available below the reviewer comments at the end of this letter.

If applicable, we recommend that you deposit your laboratory protocols in protocols.io to enhance the reproducibility of your results. Protocols.io assigns your protocol its own identifier (DOI) so that it can be cited independently in the future. For instructions see: https://journals.plos.org/plosone/s/submission-guidelines#loc-laboratory-protocols . Additionally, PLOS ONE offers an option for publishing peer-reviewed Lab Protocol articles, which describe protocols hosted on protocols.io. Read more information on sharing protocols at https://plos.org/protocols?utm_medium=editorial-email&utm_source=authorletters&utm_campaign=protocols .

We look forward to receiving your revised manuscript.

Kind regards,

Jennifer Tucker, PhD

Staff Editor

PLOS ONE

Journal Requirements:

When submitting your revision, we need you to address these additional requirements.

1. Please ensure that your manuscript meets PLOS ONE's style requirements, including those for file naming. The PLOS ONE style templates can be found at 

https://journals.plos.org/plosone/s/file?id=wjVg/PLOSOne_formatting_sample_main_body.pdf and 

https://journals.plos.org/plosone/s/file?id=ba62/PLOSOne_formatting_sample_title_authors_affiliations.pdf

2. Please expand the acronym “CIHR” (as indicated in your financial disclosure) so that it states the name of your funders in full.

This information should be included in your cover letter; we will change the online submission form on your behalf.

3. We noted in your submission details that a portion of your manuscript may have been presented or published elsewhere. [An earlier version of this research manuscript was published as a chapter in author AP's master of health science thesis document at Ontario Tech University. A copy of the thesis has been uploaded as a related manuscript file.] Please clarify whether this [conference proceeding or publication] was peer-reviewed and formally published. If this work was previously peer-reviewed and published, in the cover letter please provide the reason that this work does not constitute dual publication and should be included in the current manuscript.

4. Please include captions for your Supporting Information files at the end of your manuscript, and update any in-text citations to match accordingly. Please see our Supporting Information guidelines for more information: http://journals.plos.org/plosone/s/supporting-information. 

[Note: HTML markup is below. Please do not edit.]

Reviewers' comments:

Reviewer's Responses to Questions

**Comments to the Author**

1. Is the manuscript technically sound, and do the data support the conclusions?

The manuscript must describe a technically sound piece of scientific research with data that supports the conclusions. Experiments must have been conducted rigorously, with appropriate controls, replication, and sample sizes. The conclusions must be drawn appropriately based on the data presented.

Reviewer #1: Yes

Reviewer #2: Partly

Reviewer #3: Partly

**********

2. Has the statistical analysis been performed appropriately and rigorously?

Reviewer #1: Yes

Reviewer #2: I Don't Know

Reviewer #3: I Don't Know

**********

3. Have the authors made all data underlying the findings in their manuscript fully available?

The PLOS Data policy requires authors to make all data underlying the findings described in their manuscript fully available without restriction, with rare exception (please refer to the Data Availability Statement in the manuscript PDF file). The data should be provided as part of the manuscript or its supporting information, or deposited to a public repository. For example, in addition to summary statistics, the data points behind means, medians and variance measures should be available. If there are restrictions on publicly sharing data—e.g. participant privacy or use of data from a third party—those must be specified.

Reviewer #1: No

Reviewer #2: No

Reviewer #3: No

**********

4. Is the manuscript presented in an intelligible fashion and written in standard English?

PLOS ONE does not copyedit accepted manuscripts, so the language in submitted articles must be clear, correct, and unambiguous. Any typographical or grammatical errors should be corrected at revision, so please note any specific errors here.

Reviewer #1: Yes

Reviewer #2: Yes

Reviewer #3: Yes

**********

5. Review Comments to the Author

Please use the space provided to explain your answers to the questions above. You may also include additional comments for the author, including concerns about dual publication, research ethics, or publication ethics. (Please upload your review as an attachment if it exceeds 20,000 characters)

Reviewer #1: Thank you for the opportunity to review this interesting manuscript, which proposes a new tool for measuring sex and 4 aspects of gender in health research studies where sex/gender is not the primary focus. I appreciate this initiative as there is a lack of consensus on how to measure these constructs and a straightforward approach is most likely to be used by researchers in non-SGBA fields. I also appreciate the inclusion of multiple dimensions of gender, as the focus is often on a single aspect (e.g., identity). While I am supportive of this endeavour, I have major concerns with the assumptions and approaches taken in developing this tool, which are described below:

(1) The authors have present the gender options on a continuum between "masculine" and "feminine". I disagree with the fundamental assumption that these identities are two ends of a spectrum. Could an individual not score high on both? This format also does not capture that aspects of gender can vary significantly based on context. For example, gendered norms may dictate that a woman adopt a more feminine orientation/roles at home, but more masculine traits/roles in the workplace.

(2) No testing or validation appears to have been done purposively with individuals who have diverse gender identities. While the authors note that this is not to replace specific investigations with these populations, I would suggest adherence to the principle that designing an effective and accessible tool should begin by designing with/for the most marginalized populations, with the assumption that then it should work for everyone. This appears particularly important given the historic exclusion of gender-diverse individuals from health research, which is often attributed to measurement challenges.

(3) The demographics illustrate that it was a very homogeneous participant group with respect to ethnicity/cultural origin. In fact, in one of the supplemental files, the authors state that the most appropriate use of the SGBA-5 would be in homogenous populations. This appears somewhat problematic to me - could this approach perpetuate the general lack of diversity in health research?

(4) I'm struggling to understand the rationale of ending the Delphi study where the authors did. The gender items did not meet the a priori definition for consensus agreement, and yet they were included anyway. Did anything change over the 3 rounds? Was any feedback given that may have improved the consensus ratings? If the a priori benchmark was to find non-consensus acceptable, what was the rationale of doing the Delphi study? If this was based on inconsistencies in definitions of sex and gender, should definitions have been added to the SGBA-5 measure? I'm also concerned by the hypothesis that this was related to miscommunication on the part of the research team. This paper reads as if it is perhaps being submitted prematurely, and there is more development on the tool is first needed.

Minor comments

- I would suggest not using the term "biological sex" if what the researchers are intending to measure is "sex assigned/designated at birth", as gender-affirming medical treatments inherently change an individuals' biology.

- Does "North American" mean in the Ethnic/Cultural Origin category include Indigenous populations?

Reviewer #2: General comments:

I wanted to thank the authors for contributing to the sex and gender science. The manuscript presents the development and validation of the Sex-and Gender-Based Analysis Tool (SGBA-5) aimed at integrating sex and gender considerations into health research. This tool addresses a gap in the availability of concise, reliable, and valid measurement instruments for assessing sex and gender influences on health. The study utilizes a Delphi consensus method and a test-retest study to evaluate the tool's validity and reliability.

Below are my specific comments.

Major:

Because of the crucial role played by a tool such as this, it is essential to provide as much detail as possible in the development process. Details are needed on the methodology and results for the following steps:

- Initial Design and Development:

(1) extensive reviews of the peer-reviewed and grey literature. Providing details (or reference of a previous publication) on these are necessary, and for example, they will also allow the reviewers and readers to understand why more relevant articles were not referenced in the following statement “The four gender constructs included in the SGBA-5 were chosen because evidence was found in the literature to support their proposed pathway of health impact (gender identity,[17,18] gender expression,[19,20] gender roles,[21,22] and gendered relations[23,24]).

(2) theoretical foundation for combining a categorical measure of sex and a continuous measure of gender using 4 dimensions; the choice of 100 point-scale

- Delphi Expert Consensus: the criteria for selecting experts, demographic characteristic of participants

- Test-Retest Study: Recruitment of participants for the older adult arm

There are two main study designs in this manuscript. At least for the Delphi method, I would recommend that the authors use a reporting guideline for better structure and completeness (e.g. CREDES, Spranger J et al. 2022)

--

Introduction

-Page 5, lines 70-73, references to existing tools are needed here

Page 5, lines 74-79, Please also mentioned that a two-step measure of gender has been the recommended approach when using nominal categorizing response options (National Academies of Sciences, Engineering, and Medicine. Measuring Sex, Gender Identity, and Sexual Orientation. Washington, DC: The National Academies Press; 2022.)

Page 5, lines 79-83: the expression sex/gender has different meanings, please provide the rationale for using it here.

Methods

- Page 6, lines 100-103: I am expecting to see some references to existing tools drawn from their extensive literature review.

Discussion

Mention of the limitation regarding the method of expert selection is right; the authors would discuss the fact that the tool excludes minoritized and gender diverse people for there doesn't seem to be an option for them

Minor:

There are two main study designs in this manuscript. At least for the Delphi method, I would recommend that the authors use a reporting guideline for better structure and completeness (e.g. CREDES, Spranger J et al. 2022). The manuscript would benefit from a more structured layout E.g. Page 8, pages 134-139: I am trying to figure out what might be the best structure for this paper? I think this text should come earlier or later in the discussion.

There are minor grammatical errors and punctuation issues, e.g. "SGBA-5 tool demonstrated reliability for all items and validity of the biological sex item" could be more clearly written

Reviewer #3: the article deals with an important topic: how sex/gender can be recorded in health studies that do not primarily focus on sex/gender. To understand this more deeply, it would be helpful to explain in which aspects of health the respective dimensions surveyed can make a contribution. In my view, simply referring to studies is not sufficient to justify this.

There is also a lack of more precise information on who took part in the delphi study. Depending on who takes part in such a study will also influence the content of the feedback. Does the feedback from people who deal specifically with gender issues differ from those who do not? In my view, it would make sense to involve the expertise of gender researchers in the content of the questions and to ask health scientists about the practicability of the survey instruments.

It is precisely in the interpretation of the items that theoretical gaps appear, which again refer to my first point: Why was which gender dimension surveyed? How do these influence health? How can the milieu-specific understanding of gender be captured?

What exactly does female-male mean? These binary poles are linked to social stereotypes and ultimately reproduce them.

In order to understand how the survey instrument can be used, it is helpful not only to refer to another publication that has worked with this survey instrument. At least a brief summary would be helpful.

In my view, it cannot be concluded from the Delphi results (consensus is: no consensus!) that the gender items can be used without reservation.

**********

6. PLOS authors have the option to publish the peer review history of their article (what does this mean? ). If published, this will include your full peer review and any attached files.

If you choose “no”, your identity will remain anonymous but your review may still be made public.

**Do you want your identity to be public for this peer review?** For information about this choice, including consent withdrawal, please see our Privacy Policy .

Reviewer #1: **Yes: ** Katelynn Boerner

Reviewer #2: No

Reviewer #3: No

**********

[NOTE: If reviewer comments were submitted as an attachment file, they will be attached to this email and accessible via the submission site. Please log into your account, locate the manuscript record, and check for the action link "View Attachments". If this link does not appear, there are no attachment files.]

While revising your submission, please upload your figure files to the Preflight Analysis and Conversion Engine (PACE) digital diagnostic tool, https://pacev2.apexcovantage.com/ . PACE helps ensure that figures meet PLOS requirements. To use PACE, you must first register as a user. Registration is free. Then, login and navigate to the UPLOAD tab, where you will find detailed instructions on how to use the tool. If you encounter any issues or have any questions when using PACE, please email PLOS at figures@plos.org . Please note that Supporting Information files do not need this step.

# Author comment: Author response to Decision Letter 1

**DOI:** 10.1371/journal.pone.0323834.r003

1 Aug 2024

We want to thank the Associate Editor for providing us with the opportunity to revise and resubmit this paper. There are several instances where the reviewers have raised concerns that make assumptions about the intent of the proposed SGBA-5 measurement tool and/or the positionality of the authors of this paper that are clearly addressed in the manuscript itself. We have addressed these comments respectfully in our point by point response.

We hope that the Editorial team agrees that this tool and paper can be of significant value to the research community.

Journal Requirements:

1. Please ensure that your manuscript meets PLOS ONE's style requirements, including those for file naming. The PLOS ONE style templates can be found at https://journals.plos.org/plosone/s/file?id=wjVg/PLOSOne_formatting_sample_main_body.pdf and https://journals.plos.org/plosone/s/file?id=ba62/PLOSOne_formatting_sample_title_authors_affiliations.pdf

Thank you, we have endeavoured to update the manuscript and file names to be in accordance with these guidelines.

2. Please expand the acronym “CIHR” (as indicated in your financial disclosure) so that it states the name of your funders in full. This information should be included in your cover letter; we will change the online submission form on your behalf.

Thank you for doing so. We have updated the cover letter with the financial disclosure statement and expanded the CIHR acronym in that statement.

3. We noted in your submission details that a portion of your manuscript may have been presented or published elsewhere. [An earlier version of this research manuscript was published as a chapter in author AP's master of health science thesis document at Ontario Tech University. A copy of the thesis has been uploaded as a related manuscript file.] Please clarify whether this [conference proceeding or publication] was peer-reviewed and formally published. If this work was previously peer-reviewed and published, in the cover letter please provide the reason that this work does not constitute dual publication and should be included in the current manuscript.

A previous version of this manuscript was included as a chapter in author AP’s master’s thesis and thus is available through Ontario Tech University institutional repository of theses and dissertations. This work has not been formally published or peer-reviewed previously. We have updated the cover letter to reflect his with the following statement:

“An earlier version of this research manuscript was included as a chapter in author AP’s master’s thesis at Ontario Tech University. As part of AP’s thesis defense, the chapter was examined by AP’s thesis committee and the final thesis document is available in Ontario Tech University’s institutional repository eScholar (https://ontariotechu.scholaris.ca/). The manuscript has not been formally peer-reviewed or published and thus does not constitute dual publication.” – Cover Letter

4. Please include captions for your Supporting Information files at the end of your manuscript, and update any in-text citations to match accordingly. Please see our Supporting Information guidelines for more information: http://journals.plos.org/plosone/s/supporting-information.

Thank you, we have added the captions to the end of the manuscript and updated the file naming accordingly.

A note to all reviewers: It is important to note that the first iteration of any tool cannot be perfect. Throughout our manuscript, we emphasize that further testing is needed and that we are simply presenting a way, not the way for sex and gender-based analysis in health sciences research.

We appreciate your insights; we hope that our responses clarify the choices we have made. This tool may not be applicable in all types of research, and therefore may not be appropriate for some types of health research. We are open to new tool ideas from the research community and hope that this first version of our tool will be iterated upon.

We hope the reviewers agree that while any one tool will not be the best choice in all situations, the development of novel measurement tools like the SGBA-5 is a move in the right direction.

Reviewer #1

1. Thank you for the opportunity to review this interesting manuscript, which proposes a new tool for measuring sex and 4 aspects of gender in health research studies where sex/gender is not the primary focus. I appreciate this initiative as there is a lack of consensus on how to measure these constructs and a straightforward approach is most likely to be used by researchers in non-SGBA fields. I also appreciate the inclusion of multiple dimensions of gender, as the focus is often on a single aspect (e.g., identity). While I am supportive of this endeavour, I have major concerns with the assumptions and approaches taken in developing this tool, which are described below:

Response: Thank you for acknowledging that this is a challenging endeavour; one that many do not attempt because of the charged nature of the topic. We appreciate your comments and thoughts, and hope we have fully addressed them below.

2. The authors have present the gender options on a continuum between "masculine" and "feminine". I disagree with the fundamental assumption that these identities are two ends of a spectrum. Could an individual not score high on both? This format also does not capture that aspects of gender can vary significantly based on context. For example, gendered norms may dictate that a woman adopt a more feminine orientation/roles at home, but more masculine traits/roles in the workplace.

Response: We did not assume that the sociocultural concepts of masculinity and femininity are solely two ends of a spectrum. We state that we are using a feminine – masculine relative continuum may be a way to measure how sex and gender influences health outcomes across a sample or group.

Please see lines 99-105, and 156-164 of the manuscript as well as pages 8-9 FAQ sections: “Can the values from the SGBA-5’s gendered aspects of health items be generalized?”, and “Aren’t masculinity and femininity separate things?” of the S1 File. [SGBA-5 v1.0 documentation] for further explanation.

3. No testing or validation appears to have been done purposively with individuals who have diverse gender identities. While the authors note that this is not to replace specific investigations with these populations, I would suggest adherence to the principle that designing an effective and accessible tool should begin by designing with/for the most marginalized populations, with the assumption that then it should work for everyone. This appears particularly important given the historic exclusion of gender-diverse individuals from health research, which is often attributed to measurement challenges.

Response: One of the main strengths of this tool is that it is INCLUSIVE of gender diverse people. We have added the following lines to help clarify this:

[Methods section:]

“...however, the SGBA-5 is designed so that it can be completed by persons of sex and gender minorities as part of studies where sex or gender are not primary variables.” – Lines 169-171

[Discussion section:]

“A strength of the proposed SGBA-5 tool is that gender-diverse persons’ responses can be analyzed without necessitating the large sample sizes (often an n of 500 or more assuming a 1% proportion) that are required to analyze small-proportion nominal groups.”. – Lines 369-372

Our sample did contain gender diverse individuals. It is also important to note that the main creators of the tool are a non-binary person (AP), and a woman of colour (SD). We agree wholeheartedly with the notion of “nothing about us, without us”. This study reports the initial testing of the SGBA-5, and as such, the authors have emphasized that further testing is needed. The current recommendations to avoid using the SGBA-5 in certain situations reflect that these situations should be evaluated individually in addition to the more general validity and reliability testing processes for the new tool. Those sorts of specific use-case investigations are well outside the scope of initial scale testing. The conservative recommendations to avoid the use of a new tool in certain circumstances is a normal part of scale development and having reasonable limitations put on a measurement tool prior to those specific situations being studied should not be interpreted as that tool never being appropriate for use in those situations. Not knowing if a measurement tool is appropriate in certain situations is not the same as that tool being inappropriate for use in those situations.

4. The demographics illustrate that it was a very homogeneous participant group with respect to ethnicity/cultural origin. In fact, in one of the supplemental files, the authors state that the most appropriate use of the SGBA-5 would be in homogenous populations. This appears somewhat problematic to me - could this approach perpetuate the general lack of diversity in health research?

Response: All studies are subject to sample selection bias, and most validity/reliability studies do not make the effort to recruit multiple samples. To further clarify our recruitment and sample, we have added the following lines to the manuscript:

[Methods section, clarifying recruitment of older adults]

“...through Ontario Tech University’s Age-Friendly Campus email newsletter and through Facebook advertisements targeting older adults, 55+ years of age in the Durham Region” – Lines 256-258

[Discussion section, addressing sample homogeneity]

“Both the student and older adult samples were relatively homogenous, and thus should not be generalized to all university students or all older adults; However, the reliability results...” – Lines 412-414

Regarding the appropriate uses of the SGBA-5 in the documentation, we reiterate that not knowing if a measurement tool is appropriate in certain situations is not the same as that tool being inappropriate for use in those situations. We hope that the SGBA-5 will support more gender-based analysis in health research. The tool can be used in samples that represent a variety of cultural and ethnic backgrounds, different income levels, and more. Even if a truly random sample selection process were used, some amount homogeneity will occur in many studies samples purely as a result of non-discriminatory limiting factors (i.e., inclusion and exclusion criteria, geographic limitations, smaller sample sizes due to ethical, safety, and practicality concerns, etc.). However, just because any one sample is relatively homogenous does not mean that we as researchers are excused from our responsibility to ensure that the diversity of human health is included in our research.

5. I'm struggling to understand the rationale of ending the Delphi study where the authors did. The gender items did not meet the a priori definition for consensus agreement, and yet they were included anyway. Did anything change over the 3 rounds? Was any feedback given that may have improved the consensus ratings? If the a priori benchmark was to find non-consensus acceptable, what was the rationale of doing the Delphi study? If this was based on inconsistencies in definitions of sex and gender, should definitions have been added to the SGBA-5 measure? I'm also concerned by the hypothesis that this was related to miscommunication on the part of the research team. This paper reads as if it is perhaps being submitted prematurely, and there is more development on the tool is first needed.

Response: We ended the Delphi in accordance with pre-determined criteria as stated in Lines 237-240, methodology which is discussed on Lines 194-197. This is also explicitly discussed in the discussion section, Lines 445-448.

To clarify rationale the following lines have been added:

“The purpose of this Delphi Expert Consensus study was to receive feedback on the construct validity of the SGBA-5’s scale items from a sample of Canadian health sciences researchers (this study’s Delphi experts and the most likely initial users of the SGBA-5). This process provided evidence of each SGBA-5 scale items’ adequacy in being able to measure what that item is proposed to measure (an item’s content validity).[16,17] In accordance with threshold of evidence used to initially select the items for inclusion in the SGBA-5, it was decided a priori that any major changes (i.e., adding a new item, switching from continuous to ordinal measures, etc.) resulting from the Delphi expert’s feedback must reflect evidence in the current health sciences literature. ” – Lines 198-205

Removing scale items that were neither strongly accepted nor rejected by the Delphi Expert study when those items are grounded in literature would be dubious. This study is an initial assessment of the SGBA-5’s construct validity of health sciences researchers. Even if every item received 100% agreement, that result would only provide evidence for validity, not confirmation that scale items are entirely valid. Scale validity testing is an ongoing process requiring numerous studies (ideally conducted by independent researchers) and requires the measurement tool to be tested in multiple ways and drawing upon the expertise of many different groups. This is a normal part of scale development.

6. I would suggest not using the term "biological sex" if what the researchers are intending to measure is "sex assigned/designated at birth", as gender-affirming medical treatments inherently change an individuals' biology.

Response: Thank you for your comment. We are using this term consistently with how the SGBA-5 is designed to aid in assessing differences in health which are influenced by sex (biologically derived) and gender (socio-culturally derived). The limitations of measuring biological sex using “sex assigned at birth” are noted in Lines 131-134, and more broadly in Lines 123-127.

7. Does "North American" mean in the Ethnic/Cultural Origin category include Indigenous populations?

Response: The categories listed in the table appear as they did in the survey. Thus, it is possible that Indigenous people identified as North American. We did not have partnerships with Indigenous communities for this research. As per the First Nations principles of ownership, control, access, and possession (OCAP) principles, we cannot make any further claims related to indigenous people with this research.

Reviewer #2:

1. I wanted to thank the authors for contributing to the sex and gender science. The manuscript presents the development and validation of the Sex-and Gender-Based Analysis Tool (SGBA-5) aimed at integrating sex and gender considerations into health research. This tool addresses a gap in the availability of concise, reliable, and valid measurement instruments for assessing sex and gender influences on health. The study utilizes a Delphi consensus method and a test-retest study to evaluate the tool's validity and reliability.

Response: Thank you for appreciating the work we’ve undertaken, and for taking the time to review our manuscript. We appreciate your thoughtful comments and have addressed each of them below.

2. Because of the crucial role played by a tool such as this, it is essential to provide as much detail as possible in the development process. Details are needed on the methodology and results for the following steps:

- Initial Design and Development:

(1) extensive reviews of the peer-reviewed and grey literature. Providing details (or reference of a previous publication) on these are necessary, and for example, they will also allow the reviewers and readers to understand why more relevant articles were not referenced in the following statement “The four gender constructs included in the SGBA-5 were chosen because evidence was found in the literature to support their proposed pathway of health impact (gender identity,[17,18] gender expression,[19,20] gender roles,[21,22] and gendered relations[23,24]).

Response: We agree with the reviewer that it is critical to clearly articulate the work done to develop the tool. In the section referenced, we comment on the existing literature, and provide our references for each of the constructs. To ensure tha

Attachment Submitted filename: Response to Reviewers.docx

# Peer review: Decision Letter 1

**DOI:** 10.1371/journal.pone.0323834.r004

**Authors:** Pasyodun Koralage Buddhika Mahesh

18 Nov 2024

PONE-D-24-12283R1Initial Validity and Reliability Testing of the SGBA-5PLOS ONE

Dear Dr. Putman,

Thank you for submitting your manuscript to PLOS ONE. After careful consideration, we feel that it has merit but does not fully meet PLOS ONE’s publication criteria as it currently stands. Therefore, we invite you to submit a revised version of the manuscript that addresses the points raised during the review process.

Please submit your revised manuscript by Jan 02 2025 11:59PM. If you will need more time than this to complete your revisions, please reply to this message or contact the journal office at plosone@plos.org . When you're ready to submit your revision, log on to https://www.editorialmanager.com/pone/ and select the 'Submissions Needing Revision' folder to locate your manuscript file.

Please include the following items when submitting your revised manuscript:

- A rebuttal letter that responds to each point raised by the academic editor and reviewer(s). You should upload this letter as a separate file labeled 'Response to Reviewers'.
- A marked-up copy of your manuscript that highlights changes made to the original version. You should upload this as a separate file labeled 'Revised Manuscript with Track Changes'.
- An unmarked version of your revised paper without tracked changes. You should upload this as a separate file labeled 'Manuscript'.

If you would like to make changes to your financial disclosure, please include your updated statement in your cover letter. Guidelines for resubmitting your figure files are available below the reviewer comments at the end of this letter.

If applicable, we recommend that you deposit your laboratory protocols in protocols.io to enhance the reproducibility of your results. Protocols.io assigns your protocol its own identifier (DOI) so that it can be cited independently in the future. For instructions see: https://journals.plos.org/plosone/s/submission-guidelines#loc-laboratory-protocols . Additionally, PLOS ONE offers an option for publishing peer-reviewed Lab Protocol articles, which describe protocols hosted on protocols.io. Read more information on sharing protocols at https://plos.org/protocols?utm_medium=editorial-email&utm_source=authorletters&utm_campaign=protocols .

We look forward to receiving your revised manuscript.

Kind regards,

Pasyodun Koralage Buddhika Mahesh

Academic Editor

PLOS ONE

[Note: HTML markup is below. Please do not edit.]

Reviewers' comments:

Reviewer's Responses to Questions

**Comments to the Author**

1. If the authors have adequately addressed your comments raised in a previous round of review and you feel that this manuscript is now acceptable for publication, you may indicate that here to bypass the “Comments to the Author” section, enter your conflict of interest statement in the “Confidential to Editor” section, and submit your "Accept" recommendation.

Reviewer #1: (No Response)

Reviewer #4: (No Response)

Reviewer #5: (No Response)

**********

2. Is the manuscript technically sound, and do the data support the conclusions?

The manuscript must describe a technically sound piece of scientific research with data that supports the conclusions. Experiments must have been conducted rigorously, with appropriate controls, replication, and sample sizes. The conclusions must be drawn appropriately based on the data presented.

Reviewer #1: Partly

Reviewer #4: Partly

Reviewer #5: Yes

**********

3. Has the statistical analysis been performed appropriately and rigorously?

Reviewer #1: Yes

Reviewer #4: Yes

Reviewer #5: Yes

**********

4. Have the authors made all data underlying the findings in their manuscript fully available?

The PLOS Data policy requires authors to make all data underlying the findings described in their manuscript fully available without restriction, with rare exception (please refer to the Data Availability Statement in the manuscript PDF file). The data should be provided as part of the manuscript or its supporting information, or deposited to a public repository. For example, in addition to summary statistics, the data points behind means, medians and variance measures should be available. If there are restrictions on publicly sharing data—e.g. participant privacy or use of data from a third party—those must be specified.

Reviewer #1: (No Response)

Reviewer #4: Yes

Reviewer #5: Yes

**********

5. Is the manuscript presented in an intelligible fashion and written in standard English?

PLOS ONE does not copyedit accepted manuscripts, so the language in submitted articles must be clear, correct, and unambiguous. Any typographical or grammatical errors should be corrected at revision, so please note any specific errors here.

Reviewer #1: Yes

Reviewer #4: Yes

Reviewer #5: Yes

**********

6. Review Comments to the Author

Please use the space provided to explain your answers to the questions above. You may also include additional comments for the author, including concerns about dual publication, research ethics, or publication ethics. (Please upload your review as an attachment if it exceeds 20,000 characters)

Reviewer #1: Thank you for the opportunity to re-review this manuscript. I appreciate that further validation is needed beyond any initial tool. I would like to clarify the intention behind some of my original comments: My concern is that once a tool is available in the public domain, particularly a tool that is unlikely to be the primary measure of a study, that many researchers would use it “as is” rather than conducting their own reliability/validity research for their specific sample. This is why I suggested more purposive recruitment efforts towards marginalized/underrepresented populations in health research if the authors are indeed hoping for it to be broadly applicable across the Canadian health research landscape. I would hope that a more diverse validation/reliability study would help increase the appropriate use of the tool AND also exemplify a more inclusive way of conducting these types of studies.

The authors’ response states that “We did not assume that the sociocultural concepts of masculinity and femininity are solely two ends of a spectrum.” If that is the case, I am unclear on why it would be presented as such in the questionnaire. It is also not clear how is a score at the middle of the spectrum interpreted – does this mean the individual is high on femininity and masculinity, or low on both?

In their response letter the authors indicated that gender-diverse individuals were included in their sample, but this data does not appear to be reported anywhere in the manuscript (or do the authors mean that gender-diverse individuals were not excluded from participation but that gender identity data was not collected?). Please clarify.

Reviewer #4: General Comments:

The manuscript under review presents the development and initial validation of the SGBA-5, a tool intended to facilitate sex- and gender-based analysis (SGBA) in health sciences research. This is a valuable initiative, addressing the need for concise and reliable tools in this area, particularly for studies where sex or gender are not primary variables of interest.

The authors have provided responses to prior reviews and made significant revisions. The manuscript is generally well-structured, and the authors have done a good job of outlining the importance of their work. However, several areas still require further clarification or revision, particularly regarding the consensus-building process in the Delphi study and the tool’s applicability across diverse populations.

Major Issues:

1. Delphi Methodology and Consensus:

The authors have set a consensus threshold of 75% for the Delphi study, but the gendered items (e.g., identity, roles, expression) did not meet this threshold. The decision to include these items despite the lack of consensus is not fully justified. While the authors acknowledge that scale development is an ongoing process, additional clarity on why the Delphi process was halted after three rounds is necessary. Was there significant feedback across rounds? Were the disagreements rooted in theoretical differences or practical concerns?

Recommendation: The inclusion of gendered items requires stronger justification, and a deeper analysis of the feedback provided by the experts would be beneficial. A clearer explanation of why consensus was not achieved and how the authors plan to address this in future iterations would strengthen the paper.

2. Diversity of the Sample:

The manuscript notes that the study sample, particularly in the Delphi study, was relatively homogeneous in terms of ethnicity and cultural background. While this limitation is acknowledged, the lack of diversity in the sample may impact the tool’s broader applicability, particularly with respect to gender-diverse populations. The authors emphasize that future testing is needed in more diverse groups, but this point could be highlighted more strongly.

Recommendation: The limitations of the sample, especially in terms of gender diversity, should be more explicitly discussed. Future versions of the SGBA-5 should prioritize testing with gender-diverse individuals to ensure inclusivity and generalizability.

3. Theoretical Justification for Gender Constructs:

The manuscript introduces four gender constructs (identity, expression, roles, relations) but provides limited theoretical explanation for their selection. Although relevant references are included, a more thorough discussion of how these constructs influence health outcomes is needed. This would provide a stronger foundation for the tool’s use in health research.

Recommendation: A more detailed exploration of the theoretical basis for the gender constructs is needed. Specifically, the authors should expand on how these constructs have been operationalized in previous research and how they are expected to interact with health outcomes.

4. Terminology:

The manuscript uses the term "biological sex" throughout, although it is noted that gender-affirming treatments can alter biological characteristics. Several reviewers recommended the use of "sex assigned at birth" as a more accurate term in this context. While the authors have addressed this in part, a more inclusive approach to terminology would enhance the clarity and appropriateness of the tool.

Recommendation: Consider revising the terminology to "sex assigned at birth" to better account for gender-affirming treatments and evolving biological characteristics.

5. Reliability and Validity Testing:

The test-retest reliability results are promising, with strong coefficients for both the student and older adult arms. However, the inclusion of gendered items without full consensus from the Delphi study raises concerns about the validity of these items. Further evidence is needed to justify their inclusion.

Recommendation: Future studies should focus on re-evaluating these gender constructs, particularly in more diverse populations. Additionally, a stronger theoretical justification for the constructs would enhance confidence in their validity.

Minor Issues:

1. Grammatical and Stylistic Improvements:

While the manuscript is generally well-written, there are areas where clarity can be improved, particularly in the explanation of key concepts such as sex and gender. Several typographical errors and unclear phrasings should be addressed in a thorough revision.

Recommendation: A final proofreading and revision of key sections for clarity and consistency would improve the overall quality of the manuscript.

2. Methodology Clarification:

The inclusion of Figure 1, which illustrates the cyclical process of scale development, is helpful in clarifying the iterative nature of the tool's development. However, further detail on how feedback was integrated into the process and how decisions were made at each stage would be useful.

Recommendation: Additional detail on the methodology, particularly regarding feedback integration, would enhance the clarity of the manuscript.

3. Responses to Reviewers:

The authors have provided detailed responses to many reviewer concerns, but some issues, particularly around the Delphi consensus and sample diversity, require further elaboration. Addressing these points more thoroughly in future revisions would strengthen the manuscript.

Conclusion:

The development of the SGBA-5 is a valuable contribution to the field, offering a novel tool for integrating sex and gender considerations into health research. However, there are several areas that require further revision, particularly regarding the theoretical foundation of the tool, the diversity of the sample, and the consensus-building process in the Delphi study. With further refinement, this tool has the potential to make a significant impact on health research.

Recommendation:

Major Revisions

Reviewer #5: I Thank The authors for this interesting and timely paper on a tool for sex and gender-based analysis.

I have my concerns of using the feminine- masculine continuum, especially in expressing the gender-expression, gender-roles and gender-relations, based on my experience with the community. Since, the gender-roles and gender-relations and even expression may differ based on the context and would vary.

However, the test-re-test assessment shows that at least a part of the population (older adults and university students) identify it correctly. I wonder whether it would be the same for the working population as well.

Nevertheless, as the authors have mentioned, this is only the first assessment and further studies are required in a more generalized sample to assess its validity.

I suggest the authors to emphasize the fact for the readers that this tool needs further studies in more generalized sample(s) prior to its use for SGBA.

The corrections that the authors have made based on the other reviewers' comments/suggestions are acceptable to me.

**********

7. PLOS authors have the option to publish the peer review history of their article (what does this mean? ). If published, this will include your full peer review and any attached files.

If you choose “no”, your identity will remain anonymous but your review may still be made public.

**Do you want your identity to be public for this peer review?** For information about this choice, including consent withdrawal, please see our Privacy Policy .

Reviewer #1: No

Reviewer #4: No

Reviewer #5: **Yes: ** Dr. Ishanka Ayeshwari Talagala

**********

[NOTE: If reviewer comments were submitted as an attachment file, they will be attached to this email and accessible via the submission site. Please log into your account, locate the manuscript record, and check for the action link "View Attachments". If this link does not appear, there are no attachment files.]

While revising your submission, please upload your figure files to the Preflight Analysis and Conversion Engine (PACE) digital diagnostic tool, https://pacev2.apexcovantage.com/ . PACE helps ensure that figures meet PLOS requirements. To use PACE, you must first register as a user. Registration is free. Then, login and navigate to the UPLOAD tab, where you will find detailed instructions on how to use the tool. If you encounter any issues or have any questions when using PACE, please email PLOS at figures@plos.org . Please note that Supporting Information files do not need this step.

Attachment Submitted filename: Reviwer report 29_09_2024.docx

# Author comment: Author response to Decision Letter 2

**DOI:** 10.1371/journal.pone.0323834.r005

13 Dec 2024

EDITOR

Thank you for giving us the opportunity to further revise this paper. We have addressed each of the reviewer comments below, and hope that you agree that we have gone above and beyond in defending and describing this work simply because of the topic of the work. As we mentioned to the Editor and Reviewers in the previous round, this paper presents an initial assessment of a novel scale which includes two assessments of validity; establishment of face validity from the literature and small group feedback as well as the Delphi study which further investigates one aspect of the SGBA-5’s content validity in a sample of Canadian health researchers. This work also includes test-retest reliability studies in two distinct population groups. The scope of this paper is already broad, and we acknowledge this tool can benefit from future validity and reliability testing with more participants.

There is a significant gap in the tools available to researchers that is holding back our understanding of the influence of sex and gender on health. This tool is a first iteration that we hope will spark further advancement in the field.

Reviewer #1:

1. Thank you for the opportunity to re-review this manuscript. I appreciate that further validation is needed beyond any initial tool. I would like to clarify the intention behind some of my original comments: My concern is that once a tool is available in the public domain, particularly a tool that is unlikely to be the primary measure of a study, that many researchers would use it “as is” rather than conducting their own reliability/validity research for their specific sample. This is why I suggested more purposive recruitment efforts towards marginalized/underrepresented populations in health research if the authors are indeed hoping for it to be broadly applicable across the Canadian health research landscape. I would hope that a more diverse validation/reliability study would help increase the appropriate use of the tool AND also exemplify a more inclusive way of conducting these types of studies.

Response: Thank you, we agree with your concern. It is not practical to expect that each researcher conducts validity and reliability testing on each measurement tool they use. The testing of multiple aspects of validity and reliability properly across diverse populations is important and is a considerably larger task than the scope of a single paper. We have added the following limitation to the discussion section to address this concern:

“Further evaluation of the SGBA-5 should investigate the different aspects of scale validity and reliability across diverse population groups.” (Lines 433-445)

2. The authors’ response states that “We did not assume that the sociocultural concepts of masculinity and femininity are solely two ends of a spectrum.” If that is the case, I am unclear on why it would be presented as such in the questionnaire. It is also not clear how is a score at the middle of the spectrum interpreted – does this mean the individual is high on femininity and masculinity, or low on both?

Response: We have added a sentence to address the choice of using of the continuum in lines 150-153 of the manuscript. The sentence added reads as follows:

{previously existing text in curled brackets}.

As the CIHR Institute of Gender and Health notes in its definitions of sex and gender in the context of health research, “[gender] is not confined to a binary (girl/woman, boy/man) nor is it static; it exists along a continuum and can change over time”.[1] To attempt to capture more of this variation than nominal items alone could provide, {feminine – masculine analogue (continuous) scales were used…} (Lines 150-153)

The issue of interpretation is covered in the supplementary example analysis provided and in the FAQ section of supplementary File 1. We have provided a quick summary here as well.

Since the SGBA-5 is designed to assess group-level differences, the “score” of the continuous gender measures is interpreted as an ordinal series with the middle not assumed to be a true “0” point. In practice that means that health researchers would use statistical tests of difference like a t-test to see if participant’s gendered aspects of health item ‘scores’ differ between study groups (e.g., intervention group vs. control group, or participants with an outcome vs. participants without that outcome). This provides researchers insight into whether these gendered aspects of health could be confounding the study’s results (and ideally then spur further research specifically into how sex or gender may be affecting that area of health).

3. In their response letter the authors indicated that gender-diverse individuals were included in their sample, but this data does not appear to be reported anywhere in the manuscript (or do the authors mean that gender-diverse individuals were not excluded from participation but that gender identity data was not collected?). Please clarify.

Response: The SGBA-5 itself reports information on gender diversity across each of the 4 gendered aspects of health included. We did not also collect nominal gender identity due the exclusionary nature of disaggregation analysis in sample sizes we had, which are addressed in Lines 79-92 of the manuscript.

Reviewer #4:

General Comments:

1. The manuscript under review presents the development and initial validation of the SGBA-5, a tool intended to facilitate sex- and gender-based analysis (SGBA) in health sciences research. This is a valuable initiative, addressing the need for concise and reliable tools in this area, particularly for studies where sex or gender are not primary variables of interest.

The authors have provided responses to prior reviews and made significant revisions. The manuscript is generally well-structured, and the authors have done a good job of outlining the importance of their work. However, several areas still require further clarification or revision, particularly regarding the consensus-building process in the Delphi study and the tool’s applicability across diverse populations.

Response: Thank you for your appreciation and for the time you’ve taken to review our work.

Major Issues:

2. Delphi Methodology and Consensus:

The authors have set a consensus threshold of 75% for the Delphi study, but the gendered items (e.g., identity, roles, expression) did not meet this threshold. The decision to include these items despite the lack of consensus is not fully justified. While the authors acknowledge that scale development is an ongoing process, additional clarity on why the Delphi process was halted after three rounds is necessary. Was there significant feedback across rounds? Were the disagreements rooted in theoretical differences or practical concerns?

Recommendation: The inclusion of gendered items requires stronger justification, and a deeper analysis of the feedback provided by the experts would be beneficial. A clearer explanation of why consensus was not achieved and how the authors plan to address this in future iterations would strengthen the paper.

Response: Thank you for giving us the opportunity to further clarify this issue. The Delphi consensus threshold was set at a conservative 75%. We did this deliberately as we are creating a novel scale. The fact that our sample of experts did not arrive at the 75% approval threshold for an item does not solely reflect on the items’ construction. It is also a reflection that there is lack of consensus about how to measure how gender impacts health among experts. As we state in Lines 100-101, [the SGBA-5 aims to be] one way to conduct SGBA in health research studies based on current evidence of how sex and gender influence health but is certainly not the only way to do so. In fact, the enough of the optional comments we received during the first round were offensive and ignorant that we felt compelled as a research team to specify our positionality in the second round and clarify that we had good intentions and were researching in good faith. Understandably, we did not include those comments in this paper.

In terms of why the Delphi was halted, this is addressed in Lines 173-174 where we state that the Delphi study would be halted once researchers reach any stable consensus (including but not limited to consensus approval). Lines 213-215 then specify the Delphi’s study non-approval consensus threshold (difference in between-round CV of < .15). This threshold is also included in the foot notes of Table 2 (lines 296-297).

3. Diversity of the Sample:

The manuscript notes that the study sample, particularly in the Delphi study, was relatively homogeneous in terms of ethnicity and cultural background. While this limitation is acknowledged, the lack of diversity in the sample may impact the tool’s broader applicability, particularly with respect to gender-diverse populations. The authors emphasize that future testing is needed in more diverse groups, but this point could be highlighted more strongly.

Recommendation: The limitations of the sample, especially in terms of gender diversity, should be more explicitly discussed. Future versions of the SGBA-5 should prioritize testing with gender-diverse individuals to ensure inclusivity and generalizability.

Response: Thank you, we agree. We have added the following limitation to the discussion section:

“Further evaluation of the SGBA-5 should investigate the different aspects of scale validity and reliability across diverse population groups.” (Lines 433-445)

4. Theoretical Justification for Gender Constructs:

The manuscript introduces four gender constructs (identity, expression, roles, relations) but provides limited theoretical explanation for their selection. Although relevant references are included, a more thorough discussion of how these constructs influence health outcomes is needed. This would provide a stronger foundation for the tool’s use in health research.

Recommendation: A more detailed exploration of the theoretical basis for the gender constructs is needed. Specifically, the authors should expand on how these constructs have been operationalized in previous research and how they are expected to interact with health outcomes.

Response: We agree with the reviewer that a detailed exploration of the theory and its potential implications is important. Indeed, the lead author did this as part of their thesis. However, reporting that literature review is outside the scope of a paper reporting on the initial validity and reliability testing of a novel scale. Further justification of the items can be found in AP’s thesis, which is cited in line 83 of the manuscript, full reference on Lines 493-494).

5. Terminology:

The manuscript uses the term "biological sex" throughout, although it is noted that gender-affirming treatments can alter biological characteristics. Several reviewers recommended the use of "sex assigned at birth" as a more accurate term in this context. While the authors have addressed this in part, a more inclusive approach to terminology would enhance the clarity and appropriateness of the tool.

Recommendation: Consider revising the terminology to "sex assigned at birth" to better account for gender-affirming treatments and evolving biological characteristics.

Response: Thank you for your comment. We are using this term consistently with how the SGBA-5 is designed to aid in assessing differences in health which are influenced by sex (biologically derived) and gender (socio-culturally derived). The limitations of discussing the range of physical and physiological mechanisms through which sex can impact health solely through the lens of “sex assigned at birth” are noted in Lines 131-134, and more broadly in Lines 123-127. Furthermore, as Canadian researchers, we are aligning our work with national funding agency policies.

6. Reliability and Validity Testing:

The test-retest reliability results are promising, with strong coefficients for both the student and older adult arms. However, the inclusion of gendered items without full consensus from the Delphi study raises concerns about the validity of these items. Further evidence is needed to justify their inclusion.

Recommendation: Future studies should focus on re-evaluating these gender constructs, particularly in more diverse populations. Additionally, a stronger theoretical justification for the constructs would enhance confidence in their validity.

Response: This study represents initial investigation of a novel measurement tool, with the Delphi study specifically representing initial evaluation of content validity from the perspectives of a group of health researchers (paraphrased from Lines 194-196). Given the lack of previously validated items from which to compare the content, construct, or face validity of the novel SGBA-5 it would be inappropriate to overgeneralize the results based on a single study alone. We have noted the opportunities for future research, and agree with the reviewer that our initial reliability data are promising. Finally, we have referenced AP’s thesis for the readers who choose to learn more about this scale and topic.

Minor Issues:

7. Grammatical and Stylistic Improvements:

While the manuscript is generally well-written, there are areas where clarity can be improved, particularly in the explanation of key concepts such as sex and gender. Several typographical errors and unclear phrasings should be addressed in a thorough revision.

Recommendation: A final proofreading and revision of key sections for clarity and consistency would improve the overall quality of the manuscript.

Response: We have carefully reviewed the manuscript and hope we have identified and addressed all issues.

8. Methodology Clarification:

The inclusion of Figure 1, which illustrates the cyclical process of scale development, is helpful in clarifying the iterative nature of the tool's development. However, further detail on how feedback was integrated into the process and how decisions were made at each stage would be useful.

Recommendation: Additional detail on the methodology, particularly regarding feedback integration, would enhance the clarity of the manuscript.

Response: Thank you. We have clarified the optional feedback we received from the Delphi experts in adding the following lines to the manuscript:

“The optional feedback provided by the Delphi experts did not provide any new insights into or constructive critique of the SGBA-5.” (Lines 314-315)

[Please note: we also address this issue in our response to comment 2.]

9. Responses to Reviewers:

The authors have provided detailed responses to many reviewer concerns, but some issues, particularly around the Delphi consensus and sample diversity, require further elaboration. Addressing these points more thoroughly in future revisions would strengthen the manuscript.

Response: Thank you for your feedback, and comments above. We hope we have addressed them to your satisfaction, and that you agree the manuscript is clearer as a result.

Conclusion:

10. The development of the SGBA-5 is a valuable contribution to the field, offering a novel tool for integrating sex and gender considerations into health research. However, there are several areas that require further revision, particularly regarding the theoretical foundation of the tool, the diversity of the sample, and the consensus-building process in the Delphi study. With further refinement, this tool has the potential to make a significant impact on health research.

Response: Thank you again for taking the time to review this manuscript, and for acknowledging the importance of our work. We hope we have been able to address your concerns in our responses.

Reviewer #5:

1. I Thank The authors for this interesting and timely paper on a tool for sex and gender-based analysis.

Response: Thank you for your support and for the time you’ve taken to review our work.

2. I have my concerns of using the feminine- masculine continuum, especially in expressing the gender-expression, gender-roles and gender-relations, based on my experience with the community. Since, the gender-roles and gender-relations and even expression may differ based on the context and would vary.

Howev

Attachment Submitted filename: Response_to_Reviewers_auresp_2.docx

# Peer review: Decision Letter 2

**DOI:** 10.1371/journal.pone.0323834.r006

**Authors:** Pasyodun Koralage Buddhika Mahesh

14 Mar 2025

<div>PONE-D-24-12283R2Initial Validity and Reliability Testing of the SGBA-5PLOS ONE

Dear Dr. Putman,

Thank you for submitting your manuscript to PLOS ONE. After careful consideration, we feel that it has merit but does not fully meet PLOS ONE’s publication criteria as it currently stands. Therefore, we invite you to submit a revised version of the manuscript that addresses the points raised during the review process.

Please submit your revised manuscript by Apr 28 2025 11:59PM. If you will need more time than this to complete your revisions, please reply to this message or contact the journal office at plosone@plos.org . When you're ready to submit your revision, log on to https://www.editorialmanager.com/pone/ and select the 'Submissions Needing Revision' folder to locate your manuscript file.

Please include the following items when submitting your revised manuscript:

- A rebuttal letter that responds to each point raised by the academic editor and reviewer(s). You should upload this letter as a separate file labeled 'Response to Reviewers'.
- A marked-up copy of your manuscript that highlights changes made to the original version. You should upload this as a separate file labeled 'Revised Manuscript with Track Changes'.
- An unmarked version of your revised paper without tracked changes. You should upload this as a separate file labeled 'Manuscript'.

If you would like to make changes to your financial disclosure, please include your updated statement in your cover letter. Guidelines for resubmitting your figure files are available below the reviewer comments at the end of this letter.

If applicable, we recommend that you deposit your laboratory protocols in protocols.io to enhance the reproducibility of your results. Protocols.io assigns your protocol its own identifier (DOI) so that it can be cited independently in the future. For instructions see: https://journals.plos.org/plosone/s/submission-guidelines#loc-laboratory-protocols . Additionally, PLOS ONE offers an option for publishing peer-reviewed Lab Protocol articles, which describe protocols hosted on protocols.io. Read more information on sharing protocols at https://plos.org/protocols?utm_medium=editorial-email&utm_source=authorletters&utm_campaign=protocols .

We look forward to receiving your revised manuscript.

Kind regards,

Pasyodun Koralage Buddhika Mahesh

Academic Editor

PLOS ONE

Journal Requirements:

Please review your reference list to ensure that it is complete and correct. If you have cited papers that have been retracted, please include the rationale for doing so in the manuscript text, or remove these references and replace them with relevant current references. Any changes to the reference list should be mentioned in the rebuttal letter that accompanies your revised manuscript. If you need to cite a retracted article, indicate the article’s retracted status in the References list and also include a citation and full reference for the retraction notice.

[Note: HTML markup is below. Please do not edit.]

Reviewers' comments:

Reviewer's Responses to Questions

**Comments to the Author**

1. If the authors have adequately addressed your comments raised in a previous round of review and you feel that this manuscript is now acceptable for publication, you may indicate that here to bypass the “Comments to the Author” section, enter your conflict of interest statement in the “Confidential to Editor” section, and submit your "Accept" recommendation.

Reviewer #4: (No Response)

**********

2. Is the manuscript technically sound, and do the data support the conclusions?

The manuscript must describe a technically sound piece of scientific research with data that supports the conclusions. Experiments must have been conducted rigorously, with appropriate controls, replication, and sample sizes. The conclusions must be drawn appropriately based on the data presented.

Reviewer #4: Yes

**********

3. Has the statistical analysis been performed appropriately and rigorously?

Reviewer #4: Yes

**********

4. Have the authors made all data underlying the findings in their manuscript fully available?

The PLOS Data policy requires authors to make all data underlying the findings described in their manuscript fully available without restriction, with rare exception (please refer to the Data Availability Statement in the manuscript PDF file). The data should be provided as part of the manuscript or its supporting information, or deposited to a public repository. For example, in addition to summary statistics, the data points behind means, medians and variance measures should be available. If there are restrictions on publicly sharing data—e.g. participant privacy or use of data from a third party—those must be specified.

Reviewer #4: Yes

**********

5. Is the manuscript presented in an intelligible fashion and written in standard English?

PLOS ONE does not copyedit accepted manuscripts, so the language in submitted articles must be clear, correct, and unambiguous. Any typographical or grammatical errors should be corrected at revision, so please note any specific errors here.

Reviewer #4: Yes

**********

6. Review Comments to the Author

Please use the space provided to explain your answers to the questions above. You may also include additional comments for the author, including concerns about dual publication, research ethics, or publication ethics. (Please upload your review as an attachment if it exceeds 20,000 characters)

Reviewer #4: Thank you for your thoughtful revisions and responses to the previous round of comments. The manuscript reflects substantial effort in addressing key concerns, particularly in acknowledging the limitations of the sample diversity, providing insights into the reliability testing, and justifying the inclusion of the gendered constructs. These updates strengthen the study's foundational contribution as a preliminary step in the development and validation of the SGBA-5 tool. However, some areas still require further clarification and refinement to ensure the manuscript meets its full potential. Below are the comments and suggestions for further improvements.

General Decision: Resubmit with Minor Revisions

While the manuscript shows promise and significant progress has been made, a few remaining issues need to be addressed for clarity and to strengthen the theoretical and methodological underpinnings. The revisions required are minor and aimed at enhancing the transparency and robustness of the study.

Reviewer Comments

1. Delphi Study and Consensus

• The decision to halt the Delphi process after three rounds and include gendered items despite not meeting the 75% consensus threshold is partially justified. However, the rationale could be more robust. Specifically:

o Provide a more detailed analysis of the expert disagreements and clarify whether the non-consensus stemmed from theoretical differences or practical concerns.

o If feasible, summarize the key optional feedback received (even if it was not constructive) to illustrate the range of expert perspectives.

2. Sample Diversity

• While the manuscript acknowledges the homogeneity of the Delphi panel and test-retest populations, the implications of this limitation should be emphasized more strongly.

o For example, discuss how the findings might differ in gender-diverse or underrepresented populations and suggest specific future research directions to address this gap.

3. Theoretical Framework

• The four gender constructs (identity, expression, roles, relations) are an important aspect of the SGBA-5, yet their theoretical justification could be expanded.

o Include a brief discussion in the main text explaining why these constructs were chosen and how they influence health outcomes. While the reference to the thesis is helpful, a concise summary would provide more immediate context for readers.

4. Terminology

• The consistent use of "biological sex" aligns with Canadian research standards; however, complementing this with "sex assigned at birth" where appropriate would enhance inclusivity and align with best practices in gender-affirming contexts.

5. Clarity and Style

• While the manuscript is generally well-written, certain sections (e.g., discussion and methodology) would benefit from additional clarity. Simplify complex sentences and ensure consistent terminology throughout the text.

o A final proofreading pass is recommended to ensure grammatical accuracy and improve readability.

6. Methodology Transparency

• The integration of feedback from the Delphi study could be described in greater detail.

o For example, specify how disagreements or diverse opinions were handled in the revision process, even if optional feedback provided little constructive insight.

7. Supplementary Materials

• Key details from supplementary materials, such as the example analysis and methodological rationale, should be summarized briefly in the main text to make the paper more self-contained.

Conclusion

This manuscript represents an important step in advancing tools for SGBA in health research. With the suggested minor revisions, it will be well-positioned to make a significant contribution to the field. The transparency of the Delphi process, theoretical justification for the gender constructs, and methodological clarity will further enhance its impact and credibility.

Thank you for your continued dedication to this important work, and I look forward to reviewing the revised submission.

**********

7. PLOS authors have the option to publish the peer review history of their article (what does this mean? ). If published, this will include your full peer review and any attached files.

If you choose “no”, your identity will remain anonymous but your review may still be made public.

**Do you want your identity to be public for this peer review?** For information about this choice, including consent withdrawal, please see our Privacy Policy .

Reviewer #4: No

**********

[NOTE: If reviewer comments were submitted as an attachment file, they will be attached to this email and accessible via the submission site. Please log into your account, locate the manuscript record, and check for the action link "View Attachments". If this link does not appear, there are no attachment files.]

While revising your submission, please upload your figure files to the Preflight Analysis and Conversion Engine (PACE) digital diagnostic tool, https://pacev2.apexcovantage.com/ . PACE helps ensure that figures meet PLOS requirements. To use PACE, you must first register as a user. Registration is free. Then, login and navigate to the UPLOAD tab, where you will find detailed instructions on how to use the tool. If you encounter any issues or have any questions when using PACE, please email PLOS at figures@plos.org . Please note that Supporting Information files do not need this step.

Attachment Submitted filename: Reviewer comments_19_01_2025.pdf

# Author comment: Author response to Decision Letter 3

**DOI:** 10.1371/journal.pone.0323834.r007

28 Mar 2025

EDITOR

Thank you for the opportunity to respond to the reviewer’s comments. As you will note from our responses, the reviewer is no longer identifying new issues, rather, they are going back to issues that we have already addressed in the previous two rounds of revisions. Furthermore, they appear to have limited understanding of the methods we have employed, justified, and thoroughly explained in multiple places in the manuscript. Finally, we do not feel we can ever satisfy a reviewer’s personal preference for language and terminology. We have used terms that we believe are appropriate and are grounded in research.

We hope that you agree that the paper is ready for publication in its current form as a result of the rigorous review process it has undergone. This has been a long review process (original submission March 2024), so we look forward to a final decision on this submission soon.

REVIEWER # 4

1. Thank you for your thoughtful revisions and responses to the previous round of comments. The manuscript reflects substantial effort in addressing key concerns, particularly in acknowledging the limitations of the sample diversity, providing insights into the reliability testing, and justifying the inclusion of the gendered constructs. These updates strengthen the study's foundational contribution as a preliminary step in the development and validation of the SGBA-5 tool. However, some areas still require further clarification and refinement to ensure the manuscript meets its full potential. Below are the comments and suggestions for further improvements.

Response: Thank you for taking the time to review our work again, and for your kind words regarding our novel work.

Delphi Study and Consensus

2. The decision to halt the Delphi process after three rounds and include gendered items despite not meeting the 75% consensus threshold is partially justified. However, the rationale could be more robust. Specifically:

a. Provide a more detailed analysis of the expert disagreements and clarify whether the non-consensus stemmed from theoretical differences or practical concerns.

Response: Unfortunately, it appears there is some misunderstanding. This study asked the experts to rate the SGBA-5 items validity for use in health research on 1 to 5 Likert scale (the primary focus of this study), then experts had the option to provide qualitative comments regarding the items, some of whom did. As stated in the manuscript, the purpose of this was to provide “feedback on the construct validity”, not to provide insight on philosophical or theoretical differences of health sciences researchers.

This is certainly an area with potential for further research (as is noted in the discussion section in lines 366-375) but is outside the scope of the initial validity and reliability testing reported in this paper.

As we have acknowledged, in the manuscript “The resulting tool is proposed as one way to conduct SGBA in health research studies based on current evidence of how sex and gender influence health but is certainly not the only way to do so.”. Surely, there are a multitude of ways in which a new tool can be designed; the SGBA-5 is a result of the literature, methods, and approaches we chose to implement, all of which are evidence-based and justified throughout the manuscript.

b. If feasible, summarize the key optional feedback received (even if it was not constructive) to illustrate the range of expert perspectives.

Response: A qualitative analysis of the health researcher’s optional feedback is outside of the scope of this study. As we mentioned in the 2nd round of reviewer responses, the lines 314-315 were updated in the manuscript to clarify that no additional insight into the SGBA-5’s item’s validity was provided in the qualitative comments.

“The optional feedback provided by the Delphi experts did not provide any new insights into or constructive critique of the SGBA-5.” (Lines 314-315)

Sample Diversity

3. While the manuscript acknowledges the homogeneity of the Delphi panel and test-retest populations, the implications of this limitation should be emphasized more strongly.

a. For example, discuss how the findings might differ in gender-diverse or underrepresented populations and suggest specific future research directions to address this gap.

Response: This limitation has been addressed in response to a previous reviewer. Specifically, details can be found in the discussion section in lines 414-424.

It would be highly inappropriate of us to speculate on how the findings might differ in a gender-diverse or under-representative sample. To be clear, this tool has been explicitly designed as a secondary measure for health research in general populations (see initial design and development section of the manuscript, lines 151-157) – to conduct the initial validity and reliability tests in situations where the tool is not designed to work would be inconsistent with expectations of tool development research.

We agree that studying the SGBA-5 with these groups specifically should be done in the future. Further testing is also needed using larger, more diverse samples to study its potential generalizability (this version of the tool is not assumed to be generalizable) but such research is not a reasonable starting pointing for assessing a novel measurement item.

Theoretical Framework

4. The four gender constructs (identity, expression, roles, relations) are an important aspect of the SGBA-5, yet their theoretical justification could be expanded.

a. Include a brief discussion in the main text explaining why these constructs were chosen and how they influence health outcomes. While the reference to the thesis is helpful, a concise summary would provide more immediate context for readers.

Response: Sources in addition to the thesis work are cited throughout the initial design and development section (e.g., lines 100-113, and 128-129). Including more detailed description of the constructs is beyond what is typically included in a manuscript on testing validity and reliability. If the Editor wishes, we can add an appendix to the manuscript to add further detail.

Terminology

5. The consistent use of "biological sex" aligns with Canadian research standards; however, complementing this with "sex assigned at birth" where appropriate would enhance inclusivity and align with best practices in gender-affirming contexts.

Response: We appreciate your input on this, but have chosen to be consistent with use of our terminology to avoid confusion to the reader.

Clarity and Style

6. While the manuscript is generally well-written, certain sections (e.g., discussion and methodology) would benefit from additional clarity. Simplify complex sentences and ensure consistent terminology throughout the text.

a. A final proofreading pass is recommended to ensure grammatical accuracy and improve readability.

Response: Thank you, we have reviewed the manuscript.

Methodology Transparency

7. The integration of feedback from the Delphi study could be described in greater detail.

a. For example, specify how disagreements or diverse opinions were handled in the revision process, even if optional feedback provided little constructive insight.

Response: This has been answered in our response to question #2.

Supplementary Materials

8. Key details from supplementary materials, such as the example analysis and methodological rationale, should be summarized briefly in the main text to make the paper more self-contained.

Response: Including such details is not a typical in a manuscript on testing validity and reliability, however if the editor requests wishes it can be added to the manuscript as well. Alternatively, we can remove this supplementary material if it is distracting from the main purpose of the manuscript i.e. the initial validity and reliability.

Conclusion

9. This manuscript represents an important step in advancing tools for SGBA in health research. With the suggested minor revisions, it will be well-positioned to make a significant contribution to the field. The transparency of the Delphi process, theoretical justification for the gender constructs, and methodological clarity will further enhance its impact and credibility.

Thank you for your continued dedication to this important work, and I look forward to reviewing the revised submission.

Response: Thank you again for taking the time to review this manuscript, and for acknowledging the importance of our work. We hope we have been able to address your concerns in our responses.

Attachment Submitted filename: Response_to_Reviewers_auresp_3.docx

# Peer review: Decision Letter 3

**DOI:** 10.1371/journal.pone.0323834.r008

**Authors:** Pasyodun Koralage Buddhika Mahesh

16 Apr 2025

Initial Validity and Reliability Testing of the SGBA-5

PONE-D-24-12283R3

Dear Dr. Putman,

We’re pleased to inform you that your manuscript has been judged scientifically suitable for publication and will be formally accepted for publication once it meets all outstanding technical requirements.

Within one week, you’ll receive an e-mail detailing the required amendments. When these have been addressed, you’ll receive a formal acceptance letter and your manuscript will be scheduled for publication.

An invoice will be generated when your article is formally accepted. Please note, if your institution has a publishing partnership with PLOS and your article meets the relevant criteria, all or part of your publication costs will be covered. Please make sure your user information is up-to-date by logging into Editorial Manager at Editorial Manager®  and clicking the ‘Update My Information' link at the top of the page. If you have any questions relating to publication charges, please contact our Author Billing department directly at authorbilling@plos.org.

If your institution or institutions have a press office, please notify them about your upcoming paper to help maximize its impact. If they’ll be preparing press materials, please inform our press team as soon as possible -- no later than 48 hours after receiving the formal acceptance. Your manuscript will remain under strict press embargo until 2 pm Eastern Time on the date of publication. For more information, please contact onepress@plos.org.

Kind regards,

Pasyodun Koralage Buddhika Mahesh

Academic Editor

PLOS ONE

Additional Editor Comments (optional):

Reviewers' comments:

Reviewer's Responses to Questions

**Comments to the Author**

1. If the authors have adequately addressed your comments raised in a previous round of review and you feel that this manuscript is now acceptable for publication, you may indicate that here to bypass the “Comments to the Author” section, enter your conflict of interest statement in the “Confidential to Editor” section, and submit your "Accept" recommendation.

Reviewer #4: (No Response)

**********

2. Is the manuscript technically sound, and do the data support the conclusions?

The manuscript must describe a technically sound piece of scientific research with data that supports the conclusions. Experiments must have been conducted rigorously, with appropriate controls, replication, and sample sizes. The conclusions must be drawn appropriately based on the data presented.

Reviewer #4: Yes

**********

3. Has the statistical analysis been performed appropriately and rigorously?

Reviewer #4: Yes

**********

4. Have the authors made all data underlying the findings in their manuscript fully available?

The PLOS Data policy requires authors to make all data underlying the findings described in their manuscript fully available without restriction, with rare exception (please refer to the Data Availability Statement in the manuscript PDF file). The data should be provided as part of the manuscript or its supporting information, or deposited to a public repository. For example, in addition to summary statistics, the data points behind means, medians and variance measures should be available. If there are restrictions on publicly sharing data—e.g. participant privacy or use of data from a third party—those must be specified.

Reviewer #4: Yes

**********

5. Is the manuscript presented in an intelligible fashion and written in standard English?

PLOS ONE does not copyedit accepted manuscripts, so the language in submitted articles must be clear, correct, and unambiguous. Any typographical or grammatical errors should be corrected at revision, so please note any specific errors here.

Reviewer #4: Yes

**********

6. Review Comments to the Author

Please use the space provided to explain your answers to the questions above. You may also include additional comments for the author, including concerns about dual publication, research ethics, or publication ethics. (Please upload your review as an attachment if it exceeds 20,000 characters)

Reviewer #4: After a thorough review process spanning three rounds, I believe the manuscript is now ready to be accepted in its current form. The authors have shown commendable engagement throughout, responding clearly and constructively to the points raised by each reviewer.

• The study follows a solid methodological approach for an initial tool validation, using both a Delphi process and test-retest reliability assessment across two distinct participant groups. Their use of predefined stopping criteria and transparent reporting of limitations is appropriate and well-documented.

• The authors have made meaningful revisions across each round — refining their explanations of construct selection, clarifying their use of terms, and more openly acknowledging the limitations of their sample.

• While a few comments were repeated in the latest round, these largely reflect personal preferences or future directions rather than unresolved flaws. No new issues were raised that would require further revision.

• The work is methodologically robust and clearly reported. It makes a useful contribution to the literature on sex and gender-based analysis, particularly for researchers working in health fields where sex/gender are not primary variables

**********

7. PLOS authors have the option to publish the peer review history of their article (what does this mean? ). If published, this will include your full peer review and any attached files.

If you choose “no”, your identity will remain anonymous but your review may still be made public.

**Do you want your identity to be public for this peer review?** For information about this choice, including consent withdrawal, please see our Privacy Policy .

Reviewer #4: No

**********

# Editor report: Acceptance letter

**DOI:** 10.1371/journal.pone.0323834.r009

**Authors:** Pasyodun Koralage Buddhika Mahesh

PONE-D-24-12283R3

PLOS ONE

Dear Dr. Putman,

I'm pleased to inform you that your manuscript has been deemed suitable for publication in PLOS ONE. Congratulations! Your manuscript is now being handed over to our production team.

At this stage, our production department will prepare your paper for publication. This includes ensuring the following:

* All references, tables, and figures are properly cited

* All relevant supporting information is included in the manuscript submission,

* There are no issues that prevent the paper from being properly typeset

You will receive further instructions from the production team, including instructions on how to review your proof when it is ready. Please keep in mind that we are working through a large volume of accepted articles, so please give us a few days to review your paper and let you know the next and final steps.

Lastly, if your institution or institutions have a press office, please let them know about your upcoming paper now to help maximize its impact. If they'll be preparing press materials, please inform our press team within the next 48 hours. Your manuscript will remain under strict press embargo until 2 pm Eastern Time on the date of publication. For more information, please contact onepress@plos.org.

If we can help with anything else, please email us at customercare@plos.org.

Thank you for submitting your work to PLOS ONE and supporting open access.

Kind regards,

PLOS ONE Editorial Office Staff

on behalf of

Dr. Pasyodun Koralage Buddhika Mahesh

Academic Editor

PLOS ONE
